# Evidence for adaptive explanations of semelparity in animals

**DOI:** 10.1002/brv.70197

**Published:** 2026-07-07

**Authors:** Diana O. Fisher, Roberto Salguero‐Gómez

**Affiliations:** ^1^ School of the Environment, University of Queensland St Lucia Queensland 4072 Australia; ^2^ Department of Biology University of Oxford 11a Mansfield Rd Oxford OX1 3SZ UK

**Keywords:** life history evolution, matriphagy, reproductive death, semelparity‐iteroparity axis, semelparous, sexual selection, sperm competition

## Abstract

Semelparity, the reproductive strategy of reproducing once, is widespread but uncommon in animals. Classes of models to explain the evolution of semelparity are based either on age structure and mortality schedules – demographic models in which high post‐reproductive mortality risk favours high reproductive effort and semelparity results from survival costs of reproduction – or on optimal allocation of reproductive effort. We review and synthesise evolutionary explanations for the life history strategy of semelparity in animals. We find that semelparity has been reported in around 5000 of the one million named metazoan species (around 0.5%), and, including the 930 cephalopod species, explanations for semelparity have been proposed for around 1020 species, including 30 species with semelparity only in males. Phylogenetic regressions that can test the predictions of competing models to explain semelparity have been applied to four groups of animals: dasyurid and didelphid marsupials, salmonid fish, forficulid insects, and the spider families Nephilidae, Araneidae, Theridiidae, and Dolomedidae. These tests support the demographic model in taxa with semelparous females. The major drivers of post‐reproductive mortality risk that are associated with the evolution of semelparity in animals are risky and energetically demanding breeding migration, particularly to the deep ocean, and seasonally harsh climates in which periodic food shortage restricts reproductive opportunities. Species with semelparity only in males share a set of reproductive traits that appear contradictory. They cease sperm production at maturity, yet use fatal forms of mate‐guarding for paternity protection that suggest intense sperm competition, such as tearing off body parts to plug the female opening (spiders and insects) or catabolising muscle to fuel prolonged, repeated mating effort (mammals). Comparative tests suggest that post‐mating sexual selection under sexual conflict is the driver in animals with male‐only semelparity, consistent with reproductive effort models that require no environmental factor to raise post‐reproductive mortality. We propose a new explanation for the loss of sperm production ability at maturity in species with male‐only semelparity: that this is an adaptation to intense sperm competition associated with extreme reproductive effort in males.

## I. INTRODUCTION

### (1) Definition and criteria

We consider semelparity to be a life history strategy that is distinct from the ubiquitous variation in individual quality, environmental conditions, or chance that cause some individuals of iteroparous organisms to reproduce only once (Table 1). Semelparity and iteroparity have traditionally been treated as a dichotomy (Kirkendall & Stenseth, 1985), and some evolutionary models assess predicted fitness differences between semelparity and iteroparity (Table 2). However recent authors view these life history strategies as the outcome of trade‐offs between reproduction and survival, which reflect a continuum of life history variation along which organisms disperse or concentrate reproductive effort to varying degrees across their lives (Hughes, 2017). Facultatively semelparous species are close to the semelparous extreme, but are not constrained to concentrate reproduction in one episode. For example, male chinook salmon (*Oncorhynchus tshawytscha*) can be iteroparous if they do not migrate; some hatchery‐raised males recover after reproducing to spawn a second and even a third time (Unwin, Kinnison & Quinn, 1999). One way to assign taxa to relative positions along the iteroparity–semelparity continuum is to rank the probability of individual post‐reproductive survival (Fisher *et al*., 2013). Consistently iteroparous organisms have high probabilities of reproducing again after a bout of reproduction, facultatively semelparous organisms have low and variable mean probabilities of reproducing again (because only some individuals reproduce more than once), and obligately semelparous organisms are at the extreme semelparous end of the continuum with no chance of reproducing more than once at the population level. Obligately semelparous organisms have programmed death during or soon after reproduction. In this review the term semelparous refers to taxa near the semelparous extreme of the continuum, including those with and without programmed death.

**Table 1:** Glossary of terms related to the iteroparity–semelparity continuum in animals.

Term	Definition and application to animal taxa
Cost of reproduction	The trade‐off between age‐specific reproduction and parental survival, meaning that increased parental expenditure on current offspring reduces the probability of survival to reproduce again. Cost of reproduction is a central premise of evolutionary approaches to understanding life history variation in organisms.
Die‐off syndrome	The mechanism of programmed death in obligately semelparous male dasyurids (mammals). A sequence of physiological events triggered by testosterone at sexual maturity, enabling prolonged successive copulations and mate‐searching activity without eating or sleeping (post‐copulatory competition), and concluding with inevitable synchronous death of all males in a population at 11.5 months old, at the end of the rut (mating period). The chain of events includes uncontrolled cortisol response, immune system collapse, disintegration of muscle and collagen, and internal bleeding. Males trade‐off reproductive effort with post‐reproductive survival. Die‐off is physiologically similar to the mechanism of death in obligately semelparous Pacific salmon species of both sexes. This trade‐off is independent of the number of copulations in both dasyurids and salmon, as individuals do not die directly of exhaustion.
Iteroparity	The life history strategy whereby an organism can reproduce (Lt. *parus*: birth) multiple (Lt. *itero*: to repeat) times before dying. Iteroparous animals can have varied mating systems, including polyandry and monogamy.
Iteroparity–semelparity continuum	Covariation in life history traits arising from trade‐offs between resource allocation to concentrated or dispersed reproductive episodes (bouts) throughout adult life, and post‐reproductive survival. Iteroparous organisms distribute resources among several reproductive episodes during their lives, and semelparous organisms spend all reproductive resources in a single episode.
Mate‐guarding	A common response of males to sexual conflict. Mate‐guarding occurs when a male attempts to prevent access by rivals before, during, or after mating. Mate guarding behaviour includes remaining with the female post‐copulation and attacking rival males that approach, prolonged copulation that physically blocks other males, and plugging the female opening with a body part or a mating plug of ejaculate components.
Matriphagy	Consumption of the mother by her offspring. Matriphagy can include stages in which offspring first consume somatic tissues that do not result in immediate death of their mother, such as regurgitated gut lining, and later consume the rest of her body.
Monogamy	The mating system of mating with only one partner in a single reproductive cycle. Monogyny is the rare monogamous mating system in which a male can only copulate once in a lifetime.
Post‐copulatory sexual selection	Competition for fertilisation of an ovum that occurs between the sperm of rival males after mating. This type of selection includes sperm competition (e.g. through a race or raffle, numerical swamping mechanisms), and post‐mating sperm selection by females (e.g. cryptic female choice).
Obligate semelparity	The reproductive strategy in which the parent cannot reproduce a second time because the process of reproduction inevitably destroys its reproductive tissue or critical body parts.
Programmed death	Hard‐wired or genetically controlled death at a particular point in development. Programmed death can occur in iteroparous and semelparous organisms of either sex.
Polyandry	The mating system in which a female mates with multiple males in a single reproductive cycle (multiple mating in females).
Reproductive effort	The proportion of an organism's energy budget that is allocated to reproduction in a reproductive episode. Reproductive effort is often expressed as mass loss during reproduction.
Reproductive death	Death as a direct result of reproduction. Mechanisms include depleting critical body tissue to manufacture gametes, lethal injury necessary for successful copulation in males, matriphagy, and starvation while guarding eggs or young. Reproductive death in obligately semelparous organisms is programmed. Reproductive death in facultatively semelparous organisms need not be programmed.
Semelparity	The life history strategy whereby organisms reproduce only once. Adaptive semelparity is a life history strategy in which fitness benefits of spending all reproductive resources in a single episode/bout at the expense of post‐reproductive survival outweigh the fitness that would result from reducing effort and surviving to reproduce again. The aspect of reproduction that confers high enough fitness to make forgoing future reproduction worthwhile varies with the biology of the organism and environmental drivers. The stage of reproduction in which effort is most concentrated (where the major trade‐off with survival arises) therefore varies among semelparous organisms. In some species the major trade‐off with survival is during copulation (e.g. redback spider males). In some, the major trade‐off with survival is during gamete production (e.g. *Anguilla* spp. eels) or maternal care (e.g. female octopus), and is independent of the number of copulations. A bout or episode of reproduction is the costly reproductive period that is traded off with post‐reproductive survival.
Seasonal iteroparity	The life history strategy whereby organisms survive only 1 year and reproduce multiple times in one season.
Sexual cannibalism	One sex, usually the female of a mating pair, killing and consuming the other, which is usually the male, during or immediately after mating
Sexual conflict	The phenomenon whereby reproductive strategies that increase fitness differ between males and females. For example, polyandry might increase fitness in females through post‐copulatory mechanisms of sexual selection, but males increase their fitness by reducing or preventing polyandry in their mates *via* mate‐guarding. Sexual conflict is an inevitable part of sexual selection.

**Table 2:** Summary of major models to explain the evolution of semelparity and iteroparity and their predictions.

Author(s)	Conditions favouring semelparity	Conditions favouring iteroparity
Murphy (1968)		Higher variance in juvenile than in adult survival
Charnov & Schaffer (1973)	Poor post‐reproductive survival	
Young (1981)	High adult mortality or declining fecundity with age	
Bulmer (1985)		Fluctuating adult survival generating fluctuating juvenile survival under density dependence
Orzack & Tuljapurkar (1989)	Variability in adult survival	Stochastic environmental variability and correlation between vital rates
Charlesworth (1994)	High juvenile survival and low post‐reproductive survival under density‐dependent fecundity or density‐dependent juvenile survival	
Zeineddine & Jansen (2009)	Rapid directional change in environmental conditions	
Cushing (2020)	Intraspecific competition that increasingly reduces survival of juveniles as population density increases	
Gadgil & Bossert (1970)	Declining adult survival relative to juvenile survival	
Schaffer and Gadgil (1975)	Fitness benefits of reproduction increase disproportionately at high levels of reproductive effort, or costs of reproduction (reduction in future survival or reproduction caused by current reproduction) occur even at low levels of reproductive effort.	

### (2) The distinction between semelparity, reproductive death, and programmed death

Reproductive death occurs when an aspect of reproduction kills the organism, so death is directly linked to reproduction, and closely follows first reproduction (Kern & Gems, 2022). For example, part of the abdomen is torn off during copulation in honeybee drones (*Apis melifera*), causing lethal injury and reproductive death. Programmed death is hard‐wired (i.e. evidently genetically controlled) death at a particular point in development (Table 1). In some semelparous animals, reproductive death is programmed. For example, physiological changes at maturity cause river lampreys (*Lampetra fluviatilis*) to transfer their body's critical fat, protein, and calcium to intensive gamete production, and this massive investment in reproduction causes the parent's death (Bell, 1980). However, programmed death can also occur in iteroparous species, and the mechanism of programmed death need not be associated with reproduction. For example, many insects do not feed as adults. In short‐lived insects in the Order Ephemeroptera (mayflies), programmed death occurs in adults *via* a mechanism of starvation, because they have no mouthparts or functioning gut (Finch & Rose, 1995). Mayflies have an aquatic nymph stage for several months or years, then two instars out of water. The final instar when they are sexually mature lasts only days. However, because females may mate and lay successive clutches throughout their brief adult life, they do not show reproductive death (Clifford, 1982), although they show programmed death and they are close to the semelparity extreme of the iteroparity–semelparity continuum (Fig. 1).

**Fig. 1:**
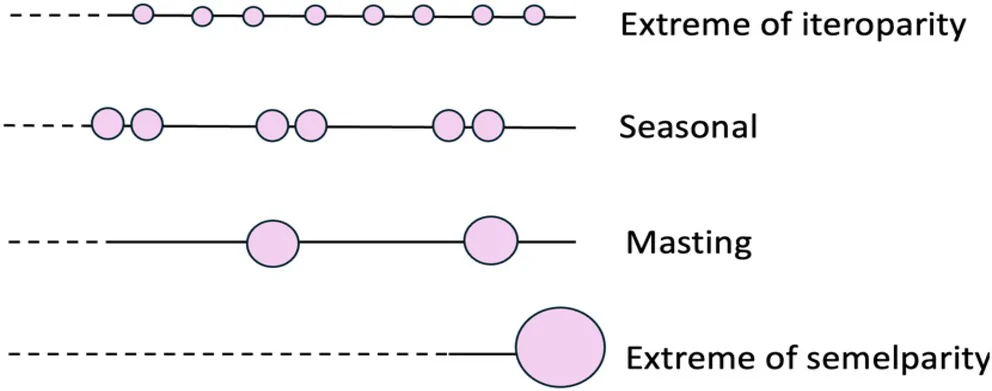
The iteroparity–semelparity continuum. Horizontal lines represent the lifetime of organisms, dashed sections correspond to the pre‐reproductive (juvenile) period. Pink dots correspond to reproductive episodes, and larger dots show higher costs of reproduction. Modified from Salguero‐Gomez *et al*. (2016). These examples represent the diversity of the iteroparity–semelparity continuum, which ranks from a single, large reproductive investment that results in parental death (extreme of semelparity; bottom), to masting, to seasonal reproduction, and last to extreme iteroparity, where the species reproduces multiple times during its lifespan, without foregoing a single reproductive opportunity.

In this review we synthesize the conclusions of comparative analyses, population comparisons, and taxon‐based studies that have investigated correlates of semelparity, including programmed reproductive death in animals. Our objective is to synthesize this research, to test predictions of competing models to explain the evolution of semelparity in animals.

### (3) Taxonomic distribution of studies

We conducted a literature review to identify metazoan taxa reported to have a semelparous life history strategy in either or both sexes by searching the Web of Science to January 2025 with the three topic search terms (‘semelpar*’ AND ‘animal’), (‘matriphag*’), and (‘sexual cannibal*’), and extracting species information from reviews (Stearns, 1976; Bell, 1980; Kirkendall & Stenseth, 1985; Finch & Rose, 1995; Hughes, 2017; Varpe & Ejsmond, 2018). We found 944 publications, listing 225 taxa (species, genera, families, tribes, and orders). To review explanations for the life history strategy of semelparity, we focused on taxa at the extreme of the iteroparity–semelparity continuum. We excluded taxa with multiple clutches in a season that are separated by several days or weeks, in which adults do not typically survive to the next year (e.g. mitten crabs *Eriochier* spp., and all frogs described as ‘virtually semelparous’ fell in this category of exclusion). We consider these to be seasonal iteroparous (Table 1). We searched the Web of Science with the search terms ‘semelpar*’ and each of the 255 taxon names, and checked the references of papers in the initial list to locate further relevant publications. We filtered the results of the above searches (1200 publications) to extract studies that offered evolutionary explanations for semelparity (i.e. fitness benefits of only reproducing once in a lifetime under particular conditions and constraints), by reading each paper (D.O.F.).

We found that semelparity has been reported in around 5000 of the one million known metazoan species‐ around 0.5% (Fig. 2). We identified publications referring to 30 named species of semelparous actinopterygiian fish, however Finch & Rose (1995) stated that around 1% of fish are likely semelparous, which would be ~2700 species. Most nudibranch molluscs are reportedly semelparous (Todd, Hadfield & Snedden, 1997), implying at least 1500 species. The annelid genus Nereis (240 species of polychaetes / ragworms) is semelparous (Finch, 1994). Males of the tardigrade genus Hypsibiidae (~300 species) are reportedly semelparous (Bertolani, 2001). We found that half of the 14 reported orders that contain semelparous taxa have at least one species with a published evolutionary explanation of the drivers and mechanisms of semelparity (Fig. 2).

**Fig. 2:**
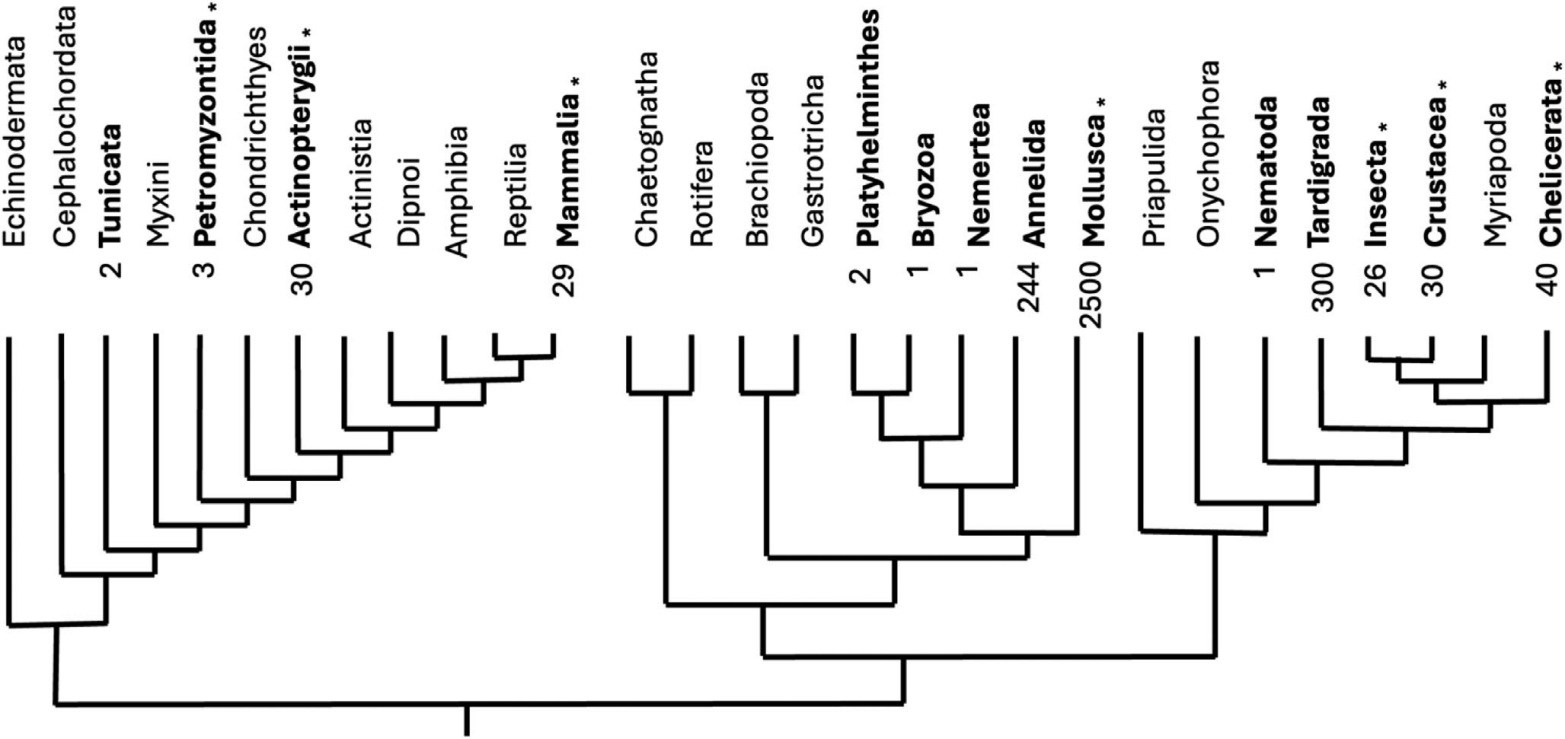
Phylogeny of metazoa (cnidaria not included) showing taxa with semelparous species (bold taxon names) and taxa in which suggested drivers and mechanisms of semelparity have been published in individual studies (asterisks). Numbers are the reported numbers of semelparous species in the taxon. Actinopterygii contains 30 species reported to be semelparous, however Finch & Rose (1995) stated that around 1% of fish are likely semelparous, which would be ~2700 species. Phylogeny based on Paps *et al*. (2023) and Laumer *et al*. (2019).

## II. DEVELOPMENT OF THEORY: EXPLANATIONS AND PREDICTORS OF SEMELPARITY

### (1) Explanations based on age structure and mortality schedules

We summarise modelled conditions that favour semelparity or iteroparity in Table 2 and Fig. 3. Using a simple approach that did not include age structure or mortality, the first quantitative model of the evolution of semelparity by Cole (1954) concluded that a very small increase in fecundity would increase fitness enough to favour semelparity over iteroparity, which suggested that most organisms should be semelparous (Cole's paradox). Murphy's (1968) model of competition without density‐dependence indicated that when conditions of environmental suitability fluctuate, in a year of low environmental suitability for juveniles that causes recruitment failure, the semelparous organism is likely to become extinct, but adults of the iteroparous organism will persist to reproduce again. That is, adult survival over multiple breeding seasons is a bet‐hedging strategy. Charnov & Schaffer (1973) resolved Cole's paradox by incorporating age class‐specific schedules of mortality to construct a demographic population model, and showed that poor post‐reproductive survival favours semelparity. Focusing on plants, Young (1981) extended the demographic model of Charnov & Schaffer (1973) to include variable intervals between bouts of reproduction and variable juvenile development time. Later analyses that have included density‐dependence, environmental variability, and mobility in models of competition between semelparous and iteroparous genotypes have resulted in less straightforward predictions. For example, Bulmer (1985) confirmed that low and variable fertility or juvenile survival favours a more iteroparous life history with a long reproductive lifespan. However fluctuating adult survival generates fluctuating juvenile survival when iteroparous genotypes are abundant, which he showed can favour iteroparity, consistent with Murphy's (1968) findings. Orzack & Tuljapurkar (1989) incorporated several sources of variation and treated the degree of semelparity as a continuous variable. They concluded that stochastic environmental variability and correlation between vital rates favours iteroparity, and variability in adult survival favours semelparity. Charlesworth (1994) also showed that high juvenile survival and low post‐breeding survival favour semelparity under density‐dependent fecundity or density‐dependent juvenile survival. However, models of Benton & Grant (1999) included density dependence and environmental stochasticity and found that reproductive effort depends on details such as which vital rates are density dependent and how these are correlated. Tesar, Kaitala & Ranta (2001) incorporated a spatial model of dispersal into Bulmer's framework with density dependence, and showed that both semelparity and iteroparity can be selected and co‐exist in the same environment. Zeineddine & Jansen (2009) found that rapid directional change in environmental conditions (as opposed to stochastic fluctuations) favours semelparity. Cushing (2020) suggested that intraspecific competition that increasingly reduces survival of juveniles as population density increases can favour the evolution of semelparity.

**Fig. 3:**
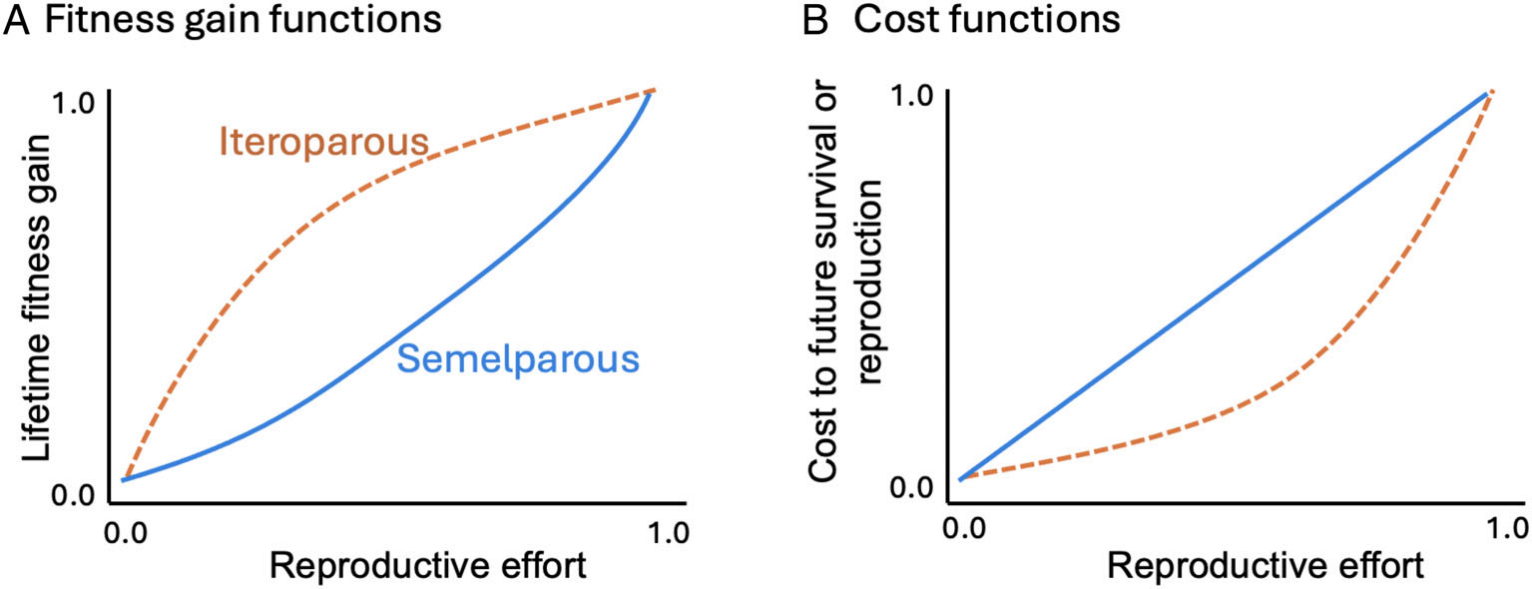
Conceptual illustration of predictions from optimal allocation models for iteroparity and semelparity. (A) Fitness gain functions describing the relationship between reproductive effort and lifetime fitness. In iteroparous taxa, fitness gains are typically convex or saturating, favouring intermediate reproductive effort, whereas in semelparous taxa fitness gains are linear or concave, such that benefits increase disproportionately at high reproductive effort. (B) Cost functions describing the effect of reproductive effort on future survival or reproduction. Iteroparous taxa show concave cost functions with relatively low initial costs, while semelparous taxa show linear or convex cost functions, incurring substantial costs even at low levels of reproductive effort. Curves are based on Gadgil & Bossert (1970) and Schaffer (1974).

Several age‐structured models have indicated that more variable environments can select against semelparity, especially when favourable environments for juveniles fluctuate more than favourable environments for adults do. However, more complex models with spatial structure, density‐dependence and dispersal show a weaker association between variation in environmental suitability and degree of semelparity. Alternative strategies that allow semelparous organisms to avoid breeding in poor seasons, allowing them to persist in variable environments include delayed reproduction and indeterminate growth (Orzack & Tuljapurkar, 1989; Benton & Grant, 1999). Iteroparity on a short time scale is another alternative adaptive response to an environment in which suitability for juveniles is variable and unpredictable.

### (2) Explanations based on optimal allocation of reproductive effort

Most demographic models address selection for the dichotomous extremes of the iteroparity–semelparity continuum based on two age categories (juvenile and adult). Another class of models is based on trade‐off curves between reproductive effort and survival at successive age classes over an organism's life. In the first of these, Gadgil & Bossert (1970) constructed graphical models of fitness gain with age based on five age classes (the contribution to fitness of the offspring produced at each age resulting from each level of reproductive effort) and based on cost (the reduction in reproductive contribution at each age caused by reduced survival, growth, and fecundity) (Fig. 3). Gadgil & Bossert (1970) realised that the optimal reproductive effort at each age class is a compromise between fitness gain and cost that depends on the shape of the gain and cost curves with effort. Iteroparity is favoured if either the gain function is convex or the cost function is concave, because intermediate reproductive effort can be optimal. They also concluded that as adult survival declines relative to juvenile survival, selection favours increasing reproductive effort at maturity. Using an optimal allocation approach with age structure, Schaffer (1974) agreed that semelparity is usually favoured when fecundity, and growth and survival are simple convex functions of effort, however alternative iteroparous life histories can evolve and multiple solutions are possible if fecundity and survival/growth vary with effort in a more complex way. The reproductive effort models of Schaffer (1974) and Schaffer & Gadgil (1975) predict that semelparity will be favoured when fitness benefits of reproduction increase disproportionately at high levels of reproductive effort, or costs of reproduction (reduction in future survival or reproduction caused by current reproduction) occur even at low levels of reproductive effort. Later optimal allocation models concluded that, if reproductive output escalates disproportionately with increasing effort, selection can also favour iteroparity with mast years, i.e. a strategy of concentrated reproduction close to the semelparity end of the spectrum (Pugliese, 1988; Klinkhamer, Kubo & Iwasa, 2008).

### (3) Predictions from theory

Three major predictions can be made regarding age structured models with environmental variability (bet hedging). The first is that semelparous taxa will occupy locations where conditions critical to juvenile survival are either stable or cyclical and predictable. Their more iteroparous relatives occur where these conditions vary less predictably. Second, semelparous taxa depend on specific resources such as food or breeding sites that are constant, or that respond strongly to predictable seasonal temperature and / or rainfall cycles. And third, in more semelparous taxa, periodic peaks in seasonal resources are associated with peaks of reproductive success.

Age structured models with differential mortality (demographic models) predict that the natural history of semelparous taxa involves an ecological or biological driver that lowers post‐reproduction survival prospects (Fig. 3). This results in organisms from these taxa having negligible probabilities of surviving to reproduce again, even if they did not allocate so much to first reproduction that they forgo all future reproduction. Their more iteroparous relatives lack this driver. Additionally, according to this type of model, semelparous organisms show greater reproductive effort than their more iteroparous relatives, and effort increases with degree of semelparity in their phylogenetic lineage.

Optimal allocation models predict, first, that the shape of the curve describing how age‐specific reproductive effort trades off with fitness over the lifetime will differ between semelparous and iteroparous taxa. In semelparous organisms the gain function (fitness *versus* age) is not convex: fitness has a concave or linear relationship with age; and the cost function (future reproduction / survival cost *versus* age) is not concave: reproductive or survival cost has a convex or linear relationship with age (Fig. 3). In semelparous organisms, fitness benefits of reproduction increase disproportionately at high levels of reproductive effort, or costs of reproduction are initially high at low levels of reproductive effort. Optimal allocation models additionally predict that semelparous organisms will show greater reproductive effort than their more iteroparous relatives. Finally, a driver of higher post‐reproductive than juvenile mortality is not necessary in this model.

Both demographic and optimal allocation models predict higher reproductive effort in semelparous taxa. However, high reproductive effort with substantial cost to future reproduction does not necessarily favour semelparity. For example, despite the fact that some species of echinoderms brood young in their mouths and do not eat for many months, none are known to be semelparous (Pandian, 2018). For instance, the Antarctic sea star *Neosmilaster georgianus* depletes its energy stores during months of brooding and takes 2 years to accumulate enough body reserves to reproduce again (Pandian, 2018). Low adult mortality after reproduction makes iteroparity viable in females of these species.

To test predictions of demographic and optimal allocation models, researchers need lifetime data on age‐specific reproduction and survival. It is obviously impossible to measure post‐reproductive survival rate and age‐specific reproductive effort in later life in species that have already evolved semelparity. Two approaches can overcome this problem: (*i*) comparison of the biology of individuals that vary in the degree of semelparity between populations or subpopulations; and (*ii*) multi‐species analysis of correlates of evolutionary changes using phylogenetic comparative methods in families that vary in the degree of semelparity among species or genera. The more powerful approach of phylogenetic comparative tests has been applied to two taxonomic groups of vertebrates, and two of arthropods.

## III. PHYLOGENETIC COMPARATIVE TESTS: FAMILIES THAT VARY AMONG SPECIES AND POPULATIONS IN DEGREE OF SEMELPARITY

### (1) Marsupial mammals: Dasyuridae and Didelphidae

Males of the 36 genera of insectivorous marsupials in the Australasian family Dasyuridae and the South American family Didelphidae span the iteroparity to semelparity continuum. Males of the dasyurid marsupial genera *Antechinus*, *Phascogale*, and *Dasykaluta* are at the extreme end of the semelparity spectrum, as they mate during a single rut over a few days at 11 months old, and die within 2–3 weeks (Fisher *et al*., 2006, 2006, 2013; Hayes *et al*., 2019). Programmed reproductive death in males has evolved twice in dasyurids (Fisher *et al*., 2013). In these genera, all reproductive males die at the end of the rut regardless of mating success or mate number, only castrated males can survive. Escalating effects of plasma corticosteroid hormones inhibit immune function and cause somatic tissue inflammation and disintegration, causing bleeding and infections. This hormonal cascade is triggered by raised testosterone at maturity, and dasyurid genera with programmed reproductive death also have programmed spermatogenic failure a month before the rut, so they rely on stored sperm in the epididymis in the rut (Kerr & Hedger, 1983). Males deplete their energy and nitrogen reserves to fuel constant activity during the rut (Woollard, 1971), and multiple successive copulations of 12–14 h each (Fisher *et al*., 2006a). During the brief rut, males use intensive strategies of mate‐guarding to physically block rivals and their sperm: long copulation, a mating tie (preventing disengagement for many hours), and a plug of congealed semen (Fisher *et al*., 2006a).

Two species of other dasyurid genera have facultatively semelparous males and lack reproductive death (the northern quoll *Dasyurus hallucatus* and the dibbler *Parantechinus apicalis*). Hormonal changes increase activity, and long, repeated copulations reduce male survival (Fisher *et al*., 2013; Edwards, Palme & Boonstra, 2016; Gaschk *et al*., 2023). In northern quolls *D. hallucatus*, male effort is associated with sleep deprivation that ultimately reduces male survival (Gaschk *et al*., 2023). The other five species of *Dasyurus*, and the other eight genera in the family have iteroparous males with varying dispersion of lifetime reproduction (Fisher *et al*., 2013). Among South American marsupials, both sexes of didelphids vary in life history strategy, but none have the extreme of obligate male semelparity and reproductive death shown in the Australian genera *Antechinus*, *Phascogale*, and *Dasykaluta* (Fisher *et al*., 2013).

Proposed adaptive explanations for semelparity and programmed death in male dasyurids have included aspects of bet‐hedging, demographic, and optimal allocation models. The seasonal constraint (bet‐hedging) hypothesis of Braithwaite & Lee (1979) proposed that male insectivorous marsupials are prone to evolve lethal competition for mates because females are constrained to leave a 12‐month gap between litters, as a peak in arthropod prey occurs annually in their seasonally predictable forest habitats. Therefore weaning success depends on this spike in food, and females have a long lactation time relative to body size (a marsupial trait). Braithwaite & Lee's explanation included an aspect of the demographic model: they argued that unspecified environmental causes of mortality result in adult males of these small mammals seldom surviving a year after maturity. Lethal competition in the first season is thus adaptive because males are unlikely to breed again. An alternative explanation by Dickman (1994) and Lazenby‐Cohen & Cockburn (1988) was that peculiarities of the mating system lead to extreme sexual selection and a trade‐off between reproductive effort and post‐mating survival in male antechinus. This is consistent with an optimal allocation model that does not require environmental causes of mortality specific to males; intense post‐mating sexual selection, such as sperm competition alone could be the driver.

Fisher *et al*. (2013) tested predictions of the seasonal bet‐hedging and optimal allocation models using phylogenetic comparative analysis of 52 species of insectivorous marsupials (dasyurids and didelphids). Supporting the seasonal constraint step of Braithwaite and Lee's seasonal bet‐hedging explanation, they showed that more semelparous species have shorter breeding periods, breeding season length declines as seasonal prey predictability increases, and late lactation and weaning (times of peak maternal energy demand and mortality risk for offspring) coincide with the month of peak leaf litter arthropod abundance in monoestrous species. Supporting a prediction of the optimal allocation model, they found that male post‐mating survival declines with increasing intensity of sperm competition indicators (testes size and copulation duration). Fisher *et al*. (2006a) showed experimentally that female brown antechinus (*Antechinus stuartii*) gain lifetime fitness benefits by mating promiscuously and escalating sperm competition among their mates, because individuals with the most competitive sperm sire offspring with the highest survival. Fertilisation success is skewed to behaviourally dominant males, which die most rapidly (Fisher *et al*., 2006b; Holleley *et al*., 2006). The escalating fitness benefits as a function of mate‐guarding effort in these semelparous dasyurids might therefore fit the prediction of the optimal allocation model, that fitness benefits of reproduction increase disproportionately at high levels of reproductive effort.

Fisher *et al*. (2006b) rejected the prediction of the demographic model that males, but not females, would have negligible post‐mating survival if they did not trade off mating effort with survival. Males and females are ecologically similar, and the sexes share home ranges and nests (Lazenby‐Cohen & Cockburn, 1988; Fisher, 2005), yet female antechinus can live 2 years and reproduce twice (Fisher & Blomberg, 2011). This lack of sex segregation in habitat indicates that no idiosyncratic environmental factor raises adult male mortality risk sufficiently after mating to drive the evolution of semelparity under predictions of the demographic model. Semelparity in male dasyurid and didelphid mammals is consistent with aspects of the bet‐hedging and optimal allocation models, not the demographic model.

### (2) Actinopterygiian fish: Salmonidae

The family Salmonidae comprises 11 genera of carnivorous northern hemisphere fish, including salmon, trout, char, grayling, and whitefish. Salmonids inhabit salt water environments such as the sea or coastal regions (anadromous species) or freshwater lakes (freshwater populations) as adults. Salmonids inhabiting lakes have lower energy expenditure and mobility than those in rivers (Lennox *et al*., 2024). The distance that species migrate from deeper water to reproduce in shallow water varies from a few hundred metres to thousands of kilometres, typically they spawn in headwaters of streams and rivers. Juveniles develop in the relatively safe shallow stream environment. Salmonid species and populations vary in degree of semelparity. Females of the five North American Pacific salmon species *Oncorhyncus kisutch* (coho), *O. tshawytscha* (chinook), *O. nerka* (sockeye), *O. gorbuscha* (pink), and *O. keta* (chum) and anandromous populations of the Asian Pacific salmon species *O. masou* (masu) are semelparous and reproductive death is programmed (Crespi & Teo, 2002; Morita *et al*., 2018). In most populations males are also semelparous, but males of some species lack programmed reproductive death: some males of lake‐inhabiting coho, chinook, sockeye, and masu can be iteroparous (Unwin *et al*., 1999; Crespi & Teo, 2002; Morita *et al*., 2018). The other six *Oncorhyncus* species including freshwater species, migratory steelhead trout *O. mykiss* and coastal cutthroat trout *O. clarkii* are iteroparous in both sexes, and Alantic salmon, trout and char (*Salmo* and *Salvelinus* spp) are iteroparous with varying dispersion of reproductive effort across the lifetime. Up to a quarter of individual Alantic salmon of both sexes are iteroparous‐ a mean of ~4% per population, and some populations are semelparous (Persson *et al*., 2023).

Programmed death in females has evolved once in salmonids – *Oncorhyncus* species with this trait are monophyletic (Kindsvater *et al*., 2016). Crespi & Teo (2002) tested predictions of the demographic model using phylogenetic comparative analysis of 12 species of anadromous salmonid species and seven freshwater populations. The hypothesized driver of semelparity in salmonids is a long, energetically costly migration against the current that raises the death rate in reproductive adults, so that adults are very unlikely to survive to a second season and successfully repeat migration (Schaffer & Elson, 1975; Evans *et al*., 2011). Habitats and mortality risks of adults and juveniles of iteroparous taxa are thus more similar to one another than in semelparous salmonid taxa. Anadromy (resulting in variation in breeding migration extent and cost) evolved twice from freshwater ancestry in salmonids (Dodson, Laroche & Lecomte, 2009); in the speciose clade comprising *Oncorhyncus, Salmo* and *Salvelinus*, and in the whitefish clade *Coregonus*. Anadromy arose during Miocene periods of glaciation, when ocean productivity increased, sea level dropped and river lengths increased, and high salmonid speciation rate was associated with colonisation of newly accessible river systems (Macqueen & Johnston, 2014), indicating that energetically demanding and physiologically stressful breeding migration drove life history evolution in salmonids, not vice versa.

Semelparous salmonids have higher reproductive investment than iteroparous species do, and proportional investment does not overlap between these two strategies: semelparous salmonids deplete more than 70% of their energy reserves during reproduction, and iteroparous species less than 60% (Fleming & Reynolds, 2004; Birnie‐Gauvin *et al*., 2021). Crespi & Teo (2002) focused on maternal investment and did not analyse male life history data. The demographic model in salmonids predicts higher fecundity, larger body size (increasing fecundity) and greater egg mass reflecting increased reproductive investment in more semelparous taxa, and that freshwater forms would be more iteroparous. Crespi & Teo's analyses supported the demographic hypothesis: freshwater salmonids that migrate short distances to reproduce were iteroparous, smaller, had smaller eggs, lower reproductive investment relative to body mass., and semelparous species with long and risky migrations from the sea to river headwaters had the largest body and egg sizes.

Kindsvater *et al*. (2016) tested quantitative predictions of the demographic model and optimal allocation using linear mixed effects and stage‐structured matrix models of 13 species and 363 populations of salmonids, including *Oncorhyncus, Salmo, and Salvelinus*. They focused on reproductive costs and extrinsic risk of mortality in females. Kindsvater *et al*. (2016) showed that semelparity has evolved in salmonids when extrinsic mortality risk is high or producing large eggs strongly reduces female survival, demonstrating that a high survival cost of migration is an extrinsic driver, and large egg size reduces a female's chance of reproducing again in semelparous salmonids. They concluded that iteroparous females evolved smaller eggs to improve maternal survival to reproduce again in safer habitats, consistent with the demographic hypothesis.

The degree of semelparity in males varies more among species and populations than it does in female salmonids, and plasma cortisol escalation is involved in programmed death in both male and female salmon and male dasyurids (Bradley, 1990; Carruth *et al*., 2000; Hruska *et al*., 2010). However, there have been no lineage‐wide comparative tests of hypotheses to explain semelparity in male salmonids. In most species migration length is the same in both sexes. Species level studies indicate that, as in females, greater investment in reproduction reduces a male's chance of spawning again in semelparous salmonids. Jonsson, Jonsson & Hansen (1997) showed that energy expenditure on migration and spawning was similar in both sexes (60–70% of body reserves) and negatively correlated with post‐spawning survival of Norwegian Atlantic salmon *S. salar*. Semelparous male sockeye salmon deplete nearly all fat and a third of their body protein during migration and spawning (Quinn & Foote, 1994). Male salmonids also have additional costs associated with reproductive competition over females and spawning sites, including investment in sexually selected traits of enlarged jaws and specialised teeth for combat, and back humps for display (Quinn & Foote, 1994). Persson *et al*. (2023) found that male Atlantic salmon lost a higher proportion of body mass between spawning events than females did. Sexual competition costs in male Atlantic salmon were higher than their costs of investment in gonad development, and the high survival cost of sexual competition was the suggested reason why males were more semelparous than females in this species (Persson *et al*., 2023). Male semelparous salmonids (chinook and sockeye) invest more in reproduction than iteroparous species do (Quinn & Foote, 1994; Unwin *et al*., 1999; Hruska *et al*., 2010). Individual male sockeye salmon that invest more and are larger‐bodied are also most likely to have priority access to nesting females, so more fertilisations (Quinn & Foote, 1994). The studies summarised above together indicate that semelparity in salmonids is consistent with predictions of the demographic model in both sexes.

### (3) Dermapteran insects: Forficulidae

The insect order Dermaptera (earwigs) originated in the tropics, and the cosmopolitan family Forficulidae comprises 65 genera worldwide (Guillet & Vancassel, 2001). All dermapterans have obligate maternal care involving the mother guarding her eggs and young, and some species provision young with food (Suzuki, 2022). Females of some forficulids have programmed semelparity because the germarium (part of the ovary) atrophies after the first clutch, others are iteroparous and raise two or more clutches in their lifetime (Guillet & Vancassel, 2001). Semelparity has evolved at least three times in female forficulids. Guillet & Vancassel (2001) constructed a molecular phylogeny of 15 European and North American dermapteran species and populations in seven genera. By mapping climate zones and life history strategies onto their phylogeny, they showed that ancestral forficulids are tropical, and semelparity arose three times in their sample, in the genera *Chelidura*, *Anechura* and *Forficula*. Each transition from the ancestral state of iteroparity to two clutches in a lifetime, and then to semelparity, accompanied diversification into a temperate then a subalpine environment, suggesting that female semelparity is an adaptation to seasonally harsh, high latitude, high elevation, cold environments. The hump earwig *Anechura harmandi*, which is restricted to alpine areas of Japan, has programmed semelparity and matriphagy [young of the first clutch eat their mothers and have poor survival if prevented (Suzuki, Kitamura & Matsubayashi, 2005)] as well as an apparent breeding migration, but not programmed reproductive death (Kohno, 1997; Suzuki *et al*., 2005). Guillet & Vancassel's comparative test supports the demographic hypothesis in which the driver of semelparity in foliculids is a seasonally harsh environment that raises the death rate in reproductive adult females, so that adults are very unlikely to survive to a second season and successfully breed again. However, male life history strategies have not been addressed, the comparative test included only seven of the 65 genera, and it omitted traits related to other key predictions that could distinguish between models in forficulids, such as greater reproductive effort in semelparous clades, and seasonal variation in critical resources for reproductive success.

### (4) Spiders: Nephilidae, Araneidae, Theridiidae, and Dolomedidae

The spider clade Entelegynae comprises 67 families. Females have two genital openings. Males have two pedipalps (the organ with which spiders transfer sperm to the female). Males of some species in the entelegyne families Nephilidae (golden orb weavers), Araneidae (orb weavers), Theridiidae (tangle‐web spiders and comb‐footed spiders) and Dolomedidae (water spiders) are semelparous. Male semelparity has evolved at least seven times in spiders (Miller, 2007; Schwartz, Wagner & Hebets, 2013, 2014) (Table 3). Mechanisms of male semelparity, including programmed death, reproductive death, sexual cannibalism and genital amputation have evolved convergently in at least three pairs of taxa in these lineages (Michalik & Rittschof, 2011).

**Table 3:** Traits and reproductive behaviour of spiders with male‐only semelparity. Ticked cells indicate presence of the trait.

Family and species	Ceases spermatogenesis at maturity	Reproductive death at 1st or 2nd insertion	Sexual cannibalism	Amputates one pedipalp at maturity	Amputates all / part of remaining pedipalp(s), which remain in female	Whole body remains in female	Insertions possible before sterility	Number of mates possible
Araneidae *Argiope aemula*	✓	✓ 2nd	✓		✓		2	1
Araneidae *A. aurantia*	✓	✓ 2nd	✓			✓	2	1
Araneidae *A. keyserlingi*	✓		✓			✓	2	1–2
Araneidae *Nephilengys borbonica*	✓		✓		✓		2	1–2
Araneidae *N. malabarensis*	✓		✓		✓		2	1–2
Nephilidae *Trichonephila fenestrata*	✓		✓		✓		2	1–2
Nephilidae *T. clavipes*	✓						2	1
Theridiidae *Echinotheridion gibberosum*	✓	✓ 1st	✓	✓	✓		1	1
Theridiidae *Tidarren argo*	✓	✓ 1st	✓	✓	✓		1	1
Theridiidae *T. cuneolatum*	✓	✓ 1st	✓	✓			1	1
Theridiidae *T. sisyphoides*	✓	✓ 1st		✓		✓	1	1
Theridiidae *Latrodectus hasselti*			✓		✓		2	1–2
Dolomedidae *Dolomedes tenebrosus*	✓	✓ 1st	✓			✓	2	1

Programmed semelparity in male spiders results from permanent spermatogenesis failure before the first mating season. For example, in the nephilid the golden orb‐web spider *Trichonephila clavipes*, testes cease producing sperm before maturity, and males rely on stored sperm on the pedipalp or in a modified seminal vesicle (Michalik, 2009; Michalik & Rittschof, 2011). Michalik (2009) and Michalik & Rittschof (2011) showed that the same mechanism of spermatogenesis failure before maturity occurs in semelparous males in the families Araneidae (*Nephilengys malabarensis* and *Argiope lobata*), Nephilidae (*Nephila senegalensis*), and Theridiidae (*Tidaren argo*). Theridiid redback spiders (*Latrodectus hasselti*) do not cease further spermatagenesis at maturity and can transfer sperm to the pedipalps repeatedly as adults (Andrade & Banta, 2002; Modanu, Michalik & Andrade, 2013). Spermatogenesis also ceases at or before maturity in males of the araneid St Andrew's cross spider *Argiope keyserlingi* (Herberstein *et al*., 2005). The semelparous male dolomedid *Dolomedes tenebrosus* shows permanent spermatogenesis failure at maturity (Linde *et al*., 2026).

A subset of species in each of these families have reproductive death in males, that is, death is a direct outcome of copulation (Miller, 2007). In the dark fishing spider *Dolomedes tenebrosus*, the Theridiids the North American comb‐footed spider *Tidarren sisyphoides* and Canary Islands comb‐footed spider *Echinotheridion gibberosum*, and the Araneid the yellow garden spider *Argiope aurantia*, males always die during sperm transfer. When the male inserts a pedipalp, the haematodochal bulb at the tip expands in the female opening, drawing the male's body fluid into the bulb, followed by spontaneous male death. Females often eat the dead male. In *D. tenebrosus*, males are always cannibalised by their mate just before or after death. In the araneid the oval St Andrew's cross spider *Argiope aemula*, the first pedipalp insertion is very brief, and males spontaneously die within a minute of inserting the second pedipalp into the same female. Copulation causes death; pulling the second pedipalp out of the female within a minute (before haematodochal bulb expansion) prevents male death (Sasaki & Iwahashi, 1995). Expansion of the haematodochal bulb in these species is permanent; the tissue distorts and hardens so each pedipalp can only be inserted in a female once (Knoflach & van Harten, 2001).

Phylogenetic comparative tests of covariates of semelparity in male spiders have not explicitly tested predictions of models to explain semelparity, but they can be interpreted in terms of these predictions. In a comparative analysis of 157 Araneoid genera and species in eight families, Miller (2007) found that sexual cannibalism is associated with genital alteration that prevents future copulations: amputation of the pedipalp and haematodochal bulb inflation, together termed genital damage. In some theridiids and araneids males can mate only once because they amputate or consume one of their two pedipalps just before maturity, or both of them during mating. For example, the Theridiid comb‐footed spiders *Tidarren sisyphoides, T. argo, T. cuneolatum*, and *Echinotheridion gibberosum* amputate one pedipalp at their penultimate molt, and the araneid hermit spiders *Nephilengys malabarensis* and *N. borbonica* amputate one or both pedipalps during mating (Knoflach & Van Harten, 2000, 2001; Knoflach & Benjamin, 2003; Ramos, Irschick & Christenson, 2004; Kuntner *et al*., 2009; Michalik & Rittschof, 2011; Lee *et al*., 2012). In *T. argo* and *E. gibberosum* the female amputates the male's remaining pedipalp during copulation, before eating him (Knoflach & van Harten, 2001) (Table 3). Using the same taxa and phylogeny as Miller (2007), Michalik & Rittschof (2011) found that ‘permanent sperm depletion’ in which males cease spermatogenesis before maturity is also associated with genital alteration and semelparity. Permanent sperm depletion, in which the lifetime supply of sperm is transferred to the pedipalps at one time, limits the number of matings (pedipalp insertions with sperm) to one or two in a lifetime (Table 3; Michalik & Rittschof, 2011).

Thus, a mechanism of reduced post‐reproductive survival in males (the female being more likely to eat the male after mating) is associated with programmed reproductive death and programmed semelparity in male spiders (Michalik & Rittschof, 2011). This mechanism could be consistent with the demographic hypothesis. Some authors have suggested that sexually cannibalistic spiders have high post‐mating mortality risk because the small males may struggle to find another mate before they die (e.g. Andrade, 1996). However, Fromhage, Elgar & Schneider (2005) showed that selection for male semelparity *via* sexual cannibalism in spiders does not need a driver of low male survival. Schneider & Fromhage (2010) pointed out that the argument that small males are unlikely to find another female after one mating is inconsistent with the intense sexual competition and adaptations associated with defence of paternity in males of these species.

If sexual cannibalism or genital mutilation increase male fitness, then sexual selection and a trade‐off between reproductive effort and post‐mating survival in male spiders with programmed semelparity could be consistent with an optimal allocation model. Intense post‐mating sexual selection is the driver, as in male dasyurids. Experimental evidence shows that lethal mate‐guarding can increase fitness in semelparous male spiders. Males of the theridiid redback spider *L. hasselti* also usually insert both pedipalps in the same female, and the tips break off in the female reducing the chance of further successful copulations by the male, and sometimes effectively blocking the entrance to that spermatheca (Andrade & Banta, 2002; Schneider & Fromhage, 2010). Male redback spiders invite sexual cannibalism by flipping into the female's mouth during copulation, and this increases their reproductive success because it increases sperm transfer duration and inhibits their mate from mating with other males. While being cannibalised, males retract vital internal organs to a protected position at the front of their body, extending their sperm transfer duration (Forster, 1992; Andrade, 1996, 2003). Sexual cannibalism and using their dead body or amputated body part as a plug are forms of mate‐guarding for paternity assurance (Table 3).

Semelparity in females involving matriphagy, lethal guarding of eggs and young, and in some taxa permanent gamete depletion (atrophy of the ovaries after one clutch) has evolved in at least five families of spiders; Araneidae, Thomisidae, Eresidae, Lycosidae, and Cheiracanthiidae (McQueen, 1978; Morse, 1994; Schneider & Lubin, 1997; Evans, 1998; Toyama, 1999; Futami & Akimoto, 2005; Salomon *et al*., 2005; Hironaka & Abe, 2012; Deshmukh, 2015; Junghanns *et al*., 2017). For example, the spider *Stegodyphus pacificus* feeds her young with regurgitated gut tissue while guarding them inside a sealed cocoon, then they consume her body (Tripathi *et al*., 2020), and the matriphagous black lace‐weaver spider (*Amaurobius ferox*) maximises her lifetime fitness if her first brood consumes her and her remaining unlaid eggs (Kim *et al*. 2000). However, female life history strategies have not been addressed using phylogenetic comparative tests. The two comparative tests of semelparity in male spiders omitted traits related to other key predictions that could distinguish between models, such as greater reproductive effort in semelparous clades, sex‐specific survival, and seasonal variation in climate and critical resources for reproductive success.

## IV. VARIATION IN BIOLOGY BETWEEN SEMELPAROUS AND ITEROPAROUS POPULATIONS AND SUB‐POPULATIONS

We are aware of three tests (one mammal and two families of marine fish) of the demographic hypothesis of semelparity in animals that are based on comparisons of populations or sexes that vary in their degree of semelparity. Demographic models predict that the biology and habitat of semelparous populations exposes them to high post‐reproductive mortality, and iteroparous populations avoid this exposure. Only one of these studies (the desert wood rat *Neotoma lepida*) modelled pre‐ and post‐reproductive mortality rates to test quantitative thresholds.

### (1) Desert wood rat *Neotoma lepida*: Cricetidae

The hypothesized driver of adult‐specific mortality in this test of the demographic hypothesis is extreme heat in the summer of some years. The populations are cohorts of a desert wood rats in Death Valley (USA). Fecundity increases with body mass in females of this species, and females were semelparous in hot years but not in cooler years. Specifically in the summer of hot years, the large body size that maximises fecundity is lethal after females raise their litter. Models showed that persistence at a smaller body size would enable survival but would result in lower lifetime reproductive output over two seasons (Smith & Charnov, 2001). Hence the demographic model explains semelparity in female desert woodrats. Fast growth to maximise fecundity caused death after first reproduction *via* overheating in an extreme desert environment, and the increase in reproduction associated with post‐reproductive female mortality was sufficient to favour semelparity (Smith & Charnov, 2001).

### (2) Actinopterygiian fish: Congridae and Osmeridae

The hypothesized driver of adult‐specific mortality in two tests of the demographic hypothesis in marine fish is an energetically expensive and physiologically stressful breeding migration to the deep ocean in only one sex, or only in some populations. Female European conger eels *Conger conger* are semelparous, and male *C. conger* are iteroparous. In this species, males permanently occupy deep ocean regions, and females migrate from shallower water to the deep ocean to spawn (Casadevall *et al*., 2017). Sex segregation in habitat, and breeding migration of female but not male conger eels are consistent with greater effort and greater post‐reproductive mortality risk in females. This divergence in both natural history and life history strategy is consistent with the demographic model, which predicts semelparity only in females (Casadevall *et al*., 2017). Energetically expensive and physiologically stressful breeding migration to the deep ocean also appears to explain divergence in life history of the osmerid capelin *Mallotus villosus*. The populations of this fish that migrate to the deep ocean to spawn are semelparous in both sexes, but beach‐spawning populations are iteroparous (Christiansen *et al*., 2008).

## V. SUGGESTED DRIVERS IN INDIVIDUAL SPECIES

### (1) Demographic model drivers suggested in studies of individual species (both sexes)

Eighty three studies suggest drivers of semelparity in 87 individual species including actinopterygiian fish, petromyzontida (lampreys), mammals (dasyurids, didelphids, and rodents), reptiles (a chameleon), chelicerata (spiders and a pseudoscorpion), insects [hymenoptera (bees and ants), hemiptera (pentatomids), dermaptera (earwigs), and mantids], crustacea (amphipods, isopods, and mysid shrimp), and nematodes (*Caenorhabditis elegans*). Additionally, a published hypothesis suggests that an increase in predation risk that occurred when their ancestors evolved soft bodies is the driver of semelparity for cephalopod molluscs in the subclass coleoidea‐the entire lineage of ~930 octopus, cuttlefish and squid (Aronson, 1991; Rocha, Guerra & Gonzalez, 2001). Semelparous female coleoid cephalopods cease feeding after laying, and brood their eggs until hatching. These hypotheses remain to be tested with clade‐wide phylogenetic regressions.

In many invertebrate groups with reported semelparous species, our literature search revealed no suggested evolutionary drivers. These include tunicates, platyhelminths, nemertea, cnidarians, annelids, tardigrades, non‐cephalopod molluscs (snails and nudibranchs), scorpions, mites, and the insect orders lepidoptera, blattoidea, orthoptera, and diptera (Fig. 2).

In 47/87 non‐cephalopods and all ~930 cephalopods (i.e. in total 96% of semelparous species) the suggested driver was a taxon‐specific cause of high mortality risk in post‐reproductive adults; the demographic model (see Section II.2, Fig. 2, Table 2). The most frequently reported driver in these species (22 species of non‐cephalopods) is a risky and arduous breeding migration at maturity, which is often against the prevailing current, because the next generation of larvae are passively transported in the opposite direction (Bell, 1980). Such migrations occur in many semelparous aquatic animals at varying scales. For example, on a smaller scale than Pacific salmon (Section III.2), the semelparous small sponge isopod, *Paracerceis sculpta*, makes a risky migration between feeding habitat (offshore algal beds) and breeding habitat (inside intertidal sponges) (Shuster, 1991). Consistent with evolution of semelparity being associated with populations and sexes of marine fish that have an arduous breeding migration to the deep ocean, the semelparous river lamprey (*Lampetra fluviatilis*) (Larsen & Dufour, 1993) and semelparous eels in the genus *Anguilla* migrate from a river to the deep ocean to spawn (Jellyman, 2021). Reproductive death occurs when females or both sexes transfer critical somatic tissue to a single bout of gamete development. *Anguilla* spp. eels, *Conger* spp. eels (except male *C. conger*, Section III.2), and lampreys invest most of their somatic tissue in migration and concurrently producing a high volume of gametes, which fills their body cavity (Larsen & Dufour, 1993; Tsukamoto *et al*., 2011; Casadevall *et al*., 2017; Jellyman, 2021).

Periodic food shortage is also a frequently‐reported driver of semelparity. We identified 11 semelparous species in which necessary resources for reproduction are restricted to a short period, and adults are reportedly unlikely to survive until the following suitable time for reproduction. In most of these species the mechanism of semelparity is matriphagy‐ females do not reproduce more than once because young consume their mother soon after her first reproductive episode. Matriphagous female spiders occur at high latitudes in the USA (*Misumena vatia*) and Japan (*Lysiteles coronatus* and *Chiracanthium japonicum*) (Futami & Akimoto, 2005; Morse, 2012). These small arthropods all experience short reproductive seasons separated by long, harsh winters. These species are suggested to be unlikely to survive to a second reproductive season, because of climate harshness and possibly also natural enemies (Morse, 1994; Morse & Stephens, 1996; Kohno, 1997; Gorokhova & Hansson, 2000; LeGrand & Morse, 2000; Guillet & Vancassel, 2001; Futami & Akimoto, 2005; Morse, 2012).

The interval between unsuitable conditions for reproduction is not necessarily seasonal in taxa that experience periodic food shortage. The neotropical pseudoscorpion (*Paratemnoides nidificator*) inhabits drought‐prone savanna, where matriphagy is triggered by food scarcity during unpredictable droughts. The reported driver of semelparity is that females are unlikely to survive to the next year that has adequate rainfall to reproduce successfully (Tizo‐Pedroso & Del‐Claro, 2005). Similarly, females of the matriphagous pentatomid bug (*Parastrachia japonensis*) provision their young with the fruit of an erratically fruiting plant species (Trumbo, 2013).

High risk of predation or parasitism from a natural enemy that efficiently targets post‐reproductive adults is also a suggested driver of female semelparity in individual studies. This can be the case even if reproduction is not punctuated by unfavourable conditions. High predation by fish on vulnerable, soft‐bodied adults has been suggested as the driver of semelparity in both sexes of ancestral coleoid cephalopods (Rocha *et al*., 2001; Ibanez *et al*., 2021). Predation or parasitism is the suggested driver in three other species of animals. The wolf spider (*Geolycosa domifex*) lays its first clutch in a sealed burrow at 3 years old, confirming that adults can survive between seasons (McQueen, 1978). Females of this species must open the burrow to release the hatchlings and, when they do, more than 99% of mothers are killed by pompilid wasps, which efficiently target breeding burrows (McQueen, 1978). Similarly, most females of the matriphagous Mediterranean spider (*Stegodyphus lineatus*) are killed by wasps, ants, and spiders while guarding the web enclosing their young from predators (Schneider & Lubin, 1997; Salomon *et al*., 2015). Iguchi & Tsukamoto (2001) suggested that predation by birds may be a driver of semelparity in ayu fish (*Plecoglossus altivelis*), and the reason why large female fish allocated up to 30% of body mass to a single spawning event, whereas small ayu spawned twice. However, no evidence of size‐specific predation was presented by the authors.

### (2) Support for sexual selection drivers in studies of individual species with male‐only semelparity

Sexual selection occurs when females choose mates, or males compete for mates. Sexual conflict, when females benefit from mating with several males, and males benefit from inhibiting multiple mating by females, is a typical outcome of female choice and male reproductive competition in animals (Table 1). In particular, mate‐guarding is male behaviour to increase the likelihood of fertilisation by their own sperm relative to that of rival males under male–male competition. Mate‐guarding includes physical proximity or body contact before or after copulation to block access by rival males, long mating duration, mechanisms to prevent the male becoming dislodged by rivals such as penis swelling, locking or holdfast structures, and mating plugs: material inserted into the female opening during or after copulation, such as congealed or hardened fluids or amputated body parts (Birkhead, 2000; Table 1). Mating plugs thus indicate sexual conflict (Birkhead, 2000). Experiments show that female antechinus gain lifetime fitness benefits by intensifying male sperm competition, *via* good genes mechanisms that increase offspring survival (Fisher *et al*., 2006a). Mate‐guarding rarely prevents further copulations, because females have evolved counter‐measures to male attempts to protect male paternity (Stockley *et al*., 2020). Sexual conflict can result in an arms race between males investing in mating plugs, female adaptations to remove and circumvent these, and males evolving counter‐measures to female resistance and further investment in mating plugs (Birkhead, 2000). Mating plugs do not need to prevent further matings to be strongly favoured by selection. They increase male fitness by delaying access by rivals, preventing females ejecting sperm, and aiding in sperm transport, raising fertilisation success of the plug‐producing male (Birkhead, 2000; Stockley *et al*., 2020).

Individual studies of bees (Baer, 2005), ants (Monnin, 1998), and mantids (Hurd, 1989; Brown & Barry, 2016) agree with phylogenetic comparative tests of dasyurids and spiders (Sections III.1 and III.4) that the driver of male‐only semelparity is intense post‐copulatory sexual selection. Mechanisms of reproductive death and programmed death show that sexual selection is connected to the evolutionary causes of semelparity in these animals; in all of these studies the mechanism of male death occurred during, or closely following mating, and is associated with mate‐guarding to protect paternity.

We identified three reported mechanisms of mate‐guarding (Table 1) in arthropods that have semelparous males and iteroparous females. The first, most common mechanism of mate guarding (11 out of 30 species, 37%), is a physical plug in which males die during copulation and either their entire body or a broken‐off section of pedipalp plugs the female opening. In this way, these species with male‐only semelparity concentrate reproductive effort to increase fertilisation success at the cost of post‐mating survival. In spiders that have a mating plug consisting of part of their body, the physical block delays but does not necessarily prevent further copulations, as the female is able to remove the whole or dismembered dead male (Kuntner *et al*., 2009). Evolution of lethal investment in reproduction does not mean that males have the upper hand in this sexual conflict; evolution of counter‐measures is ongoing. For example, in male dark fishing spiders the female consumes the dead male, removing the plug (Schwartz, Wagner & Hebets, 2016) and hermit spider mating plugs inhibit competitors briefly until the female removes them (Kuntner *et al*., 2009; Table 3). The endophallus (the male intromittant organ in hymenopterans) of a European honeybee drone *Apis mellifera* tears off during copulation, killing the male and leaving a complex plug structure lodged in the female – but still females can and typically do continue to mate with additional males (Baer, 2005). In the Yemeni spider *Tidarren argo*, males have evolved a counter measure to female plug removal: a holdfast structure on the end of the pedipalp, which delays removal by females for 4 h while continuing to deliver sperm (Knoflach & van Harten, 2001). In the harvest ant *Pogonomyrmex barbatus* and the Giant Amazonian ant (*Dinoponera quadriceps*), the female cuts off the attached male by biting through his abdomen, and further copulations are delayed while she removes the endophallus from her genital tract, which can take 30 min in *D. quadruceps* (Holldobler, 1976; Monnin, 1998).

The second, less common mechanisms of mate‐guarding in arthropods with male‐only semelparity consists of not (or not only) plugging the female opening, but inhibiting her from mating again by a mechanism of sexual cannibalism: females can be inhibited from mating with a rival male while they consume their mate. Sexual cannibalism is common in many lineages of spiders (Elgar & Schneider, 2004). Males of some taxa have evolved behaviour that capitalises on the tendency of females to cannibalise mating males, to increase their own paternity. In agreement with phylogenetic comparative tests of spiders (see Section III.4), individual studies of spiders and mantids suggest that males of some species with male‐only semelparity (eight known species) appear to use sexual cannibalism as a form of mate‐guarding, including redback spiders (see Section III.4). In the Chinese mantid *Tenodera sinensis* sexual cannibalism also benefits males that have a low chance of mating again, and females increase offspring production through improved nutrition by consuming more mates (Brown & Barry 2016).

The third mechanism of mate‐guarding in arthropods with male‐only semelparity involves physical guarding through constant proximity, and this is associated with male starvation. For example, male golden orb weavers *Trichonephila clavipes* often starve while guarding a female on her web (Cohn & Christensen, 1988). Male hermit spiders *Nephilengys malabarensis* and *N. borbonica* that survive copulation and are not cannibalised also remain on the female's web rather than leaving to feed, and they aggressively guard her from rival males for as long as they survive after mating (Kuntner *et al*. 2009).

Sexual conflict over the number of mates that females have can greatly escalate male effort and cost, consistent with the reproductive effort model. In the above individual studies, and in the comparative tests outlined in Sections III.1 and III.4, semelparous males trade off survival and future reproduction for paternity, and some males have evolved counter‐measures to inhibit subsequent competitors, yet females still circumvent male attempts at paternity assurance.

## VI. WHY DO SPECIES WITH MALE‐ONLY SEMELPARITY CEASE SPERMATOGENESIS AT MATURITY?

The shared trait of programmed loss of spermatogenesis before the mating period in the diverse taxa with male‐only semelparity is striking. In both mammals and arthropods, this loss always occurs at or before sexual maturity, well before any obvious trade‐off between reproduction and survival. For example, in male dasyurid marsupials of the genera *Antechinus* and *Phascogale* permanent spermatogenesis failure occurs at maturity, a month before the mating period, and males die 2 weeks after mating (Kerr & Hedger, 1983). Honeybees cease spermatogenesis before maturity, well before mating (Baer, 2005). Loss of spermatogenesis capacity at or just before maturity is the strongest correlate of male‐only semelparity in spiders; it characterises all such species except redback spiders (Table 3). Spiders in the genera *Tidarren* and *Echinotheridion* also amputate one of their two pedipalps before sexual maturity (emasculation, Knoflach & van Harten, 2001; Michalik & Rittschof, 2011). Curtailing sperm production seems counter‐intuitive in these animals that show such obvious adaptations to sperm competition, such as mate‐guarding behaviour, structures to physically plug the female opening after mating, and large testes.

Some authors have proposed that the function of emasculation and spermatogenesis failure in these diverse taxa is to save energy, accelerate maturity, or to increase endurance or agility for an advantage in pre‐mating sexual competition (Knoflach & Van Harten, 2000, 2001; Ramos *et al*., 2004; Michalik & Rittschof, 2011; Lee *et al*., 2012). These proposed functions do not explain the strong association of spermatogenesis failure with male‐only semelparity and the consistent timing of spermatogenesis failure abruptly at maturity, before the mating period. We suggest a semelparity‐specific adaptive function of the delay between spermatogenesis and copulation. Semelparous males transfer their lifetime supply of sperm from the testes to a storage location‐ the epididymis (mammals), seminal vesicle (hymenopterans), or pedipalp (spiders) until mating (Taggart & Templesmith, 1991; Baer, 2005; Michalik & Rittschof, 2011). Except in the dark fishing spider *D. tenebrosus*, male spiders that die spontaneously on their first insertion amputate the other pedipalp, which will never be used, ensuring that all sperm are transferred at once in one pedipalp (Ramos *et al*., 2004; Michalik, 2009) (Table 3). Sperm volume in the seminal vesicle and on the pedipalp has been measured in In *T. argo*, and shown to be exceptionally high and sufficient to entirely fill the storage organ of the female in the single transfer event, potentially leaving no room for a subsequent rival's sperm (Michalik *et al*., 2010). Spermatogenesis failure at maturity, before any sperm have been ejected from the male enables the individual's entire accumulated sperm supply to be transferred all at once. We propose that this convergent phenomenon is a male response to sperm competition. We suggest that the mechanism is analogous to capital breeding in females, in which a female can transfer energy to offspring in a single pulse by accumulating and storing fat prior to reproduction (Stephens *et al*., 2009). By ceasing spermatogenesis at maturity, semelparous males can maximise their chance of out‐competing rivals in a single intense bout of sperm competition. Depleting body tissue to fuel intense mating activity might also compromise continued sperm production and quality if the male were to maintain spermatogenesis during the mating period. Sexual conflict in which females favour escalating investment in post‐mating sexual selection is consistent with the reproductive effort model to explain male‐only semelparity (Fig. 1).

## VII. REVERSAL OF SEMELPARITY

G.C. Williams, quoted in Stearns (1976), suspected that once programmed semelparity has evolved it ought to be irreversible. This suspicion has proven to be incorrect: semelparity is evolutionarily reversible. The biological mechanisms to reverse semelparity are in fact straightforward. In animals, one evolutionary pathway to reverse semelparity for animal species that have lost the capacity to pause reproduction seasonally and regenerate their gonads more than once is to split the gametes from their one cycle of gametogenesis into a series of batches. Each batch is then a distinct episode of reproduction spread out in time, reversing the costly concentration of effort and subsequent trade‐off with post‐reproductive survival that defines adaptive semelparity (Table 1). Semelparous females that have lost the ability to ovulate more than once can split their one clutch into multiple spawning events (Grearson *et al*., 2021), and males that have lost the ability to produce more than one batch of sperm because their testes cannot regenerate can allocate their finite store of sperm to a series of matings. For example, the golden orb‐web spider *Nephila senegalensis* has reversed emasculation and sexual cannibalism, which occur in other species in the clade, but spermatogenesis still ceases with maturation. This species re‐evolved iteroparity in males by partitioning the sperm supply from their deferent ducts to their two pedipalps, to allocate to up to four females (four matings at different times over the season) (Schneider & Michalik, 2011).

Such reversals have happened multiple times in octopus and squid. Cephalopods have an optic gland that has similar function to the vertebrate pituitary. In females, the mechanism of brooding behaviour and tissue disintegration associated with semelparity depends on secretion of hormones by this gland (Wang & Ragsdale, 2018). Optic gland removal causes both sexes to resume feeding and live a few months longer, but not breed again (Wodinsky, 1977). Female octopus cannot regenerate their ovaries. However, although the ancestors of the ovoviparous two‐spot octopus *Octopus chierchiae* were semelparous (Grearson *et al*., 2021; Ibanez *et al*., 2021), this species splits a single clutch of oocytes into batches that mature at different times, and distribute these across a spawning period (Grearson *et al*., 2021). In octopus (Superorder Octopodiformes in the subclass Coleoidea) such clutch‐splitting appears to have evolved in two tropical clades during the Cenozoic (Ibanez *et al*., 2021). Most species of the diverse, globally distributed Superorder Decapodiformes (squids and sepiids) evolved from clutch‐splitting (‘asynchronous ovulating’) ancestors in a separate clade from that of ancestral Octopodiformes, and most squid retain the strategy of multiple spawning in their single breeding season (Ibanez *et al*., 2021).

The combination of multiple independent origins and losses of semelparity in diverse animal lineages potentially gives us powerful methods to test hypotheses of the evolution of semelparity using phylogenetic regressions. Variation in species position on the semelparity–iteroparity continuum has been documented in clades throughout the animal kingdom, but corrrelates that can distinguish between the predictions of competing hypotheses to explain semelparity have so far been partially investigated, in only four groups of animals.

## VIII. OUTSTANDING QUESTIONS AND FUTURE DIRECTIONS

Explicit tests of the predictions of life history theory for semelparity are much rarer than are tests of explanations for the slow–fast continuum. Few studies of animals have asked the questions that we present in Section II.3 (predictions from theory). Answers to these questions can tell us which other taxa conform to the predictions of the three main models (bet‐hedging, optimal allocation, and the demographic model). To our knowledge, no thorough tests of the predictions of the reproductive effort hypothesis have used empirical data on animals, and the only systems claimed to meet the criteria for this driver appear to be in plants (Young & Augspurger, 1991). Applying the questions in Section II.3 to more animals would confirm if animals separate neatly into three separate models. We found published comparative tests to investigate drivers of semelparity in only four animal lineages. These had gaps, because none were explicitly framed as systematic tests of the theoretical models discussed above, so each study only addressed some of the predictions. For example, we lack comparative tests of theory for male salmonids, comparative tests of spiders did not include environmental drivers or measures of reproductive effort, and comparative tests of forficulids did not include males or environmental seasonality. Another gap concerns the mechanistic, genetic, and selection pressure links between male and female life history in semelparous species. No comparative tests have included both sexes. Studies of taxa with semelparity in both sexes have focused strongly on females. Major unanswered questions include: what are the drivers of semelparity in males of taxa that show semelparity in both sexes? And are these drivers distinct from those in lineages with male‐only semelparity? Three taxonomic groups have independently evolved male‐only semelparity: spiders, dasyurid marsupials, and hymenopterans. We lack comparative tests and theoretical models to explain the evolution of semelparity in hymenopterans. Kin selection and the genetics and sex ratios of haplodiploid insects would be relevant for such models.

Study systems that could be used to confirm the generality of explanations discussed in this review, and to potentially reveal new mechanisms, include large marine clades. Particularly, comparative and experimental tests of hypotheses in both sexes of molluscs would be illuminating. Molluscs represent a globally distributed and diverse taxon, including semelparous nudibranchs, octopus and squid, and others. In most animal lineages, semelparity is scattered across recently‐evolved branches at the family or genus level suggesting that semelparous clades may not be as likely to persist as iteroparous clades are Molluscs include both ancient and recent life history transitions to semelparity in both sexes. Given that frequent extinction of semelparous clades (especially those with male‐only semelparity) is a logical reason for their scarcity and brevity in the tree of life, another outstanding question that could be answered by investigating molluscs together with other clades is the viability of semelparity on an evolutionary time scale. Is obligate semelparity an evolutionary dead end, if so, when and why? Tests of theory predictions on some other large clades with likely repeated evolution of semelparity, such as annelids (both sexes), Diptera (males) and tardigrades (males), would also be informative.

## IX. CONCLUSIONS

(1) Phylogenetic comparative tests and investigations of life history variation among populations in contrasting environments suggest that most animals are likely to follow predictions of the demographic model (Charnov & Schaffer, 1973). The demographic model is based on age structure and mortality schedules, and predicts that low probability of post‐reproductive survival favours an adaptive strategy of semelparity.
(2) Major causes of low post‐reproductive survival associated with selection for semelparity are arduous breeding migration, especially to ocean depths, and seasonally harsh environments in which conditions suitable for reproduction are punctuated by hostile conditions, especially in high latitude alpine regions and arid climates.
(3) In a small minority of semelparous animals in which only the males of a species are semelparous, comparative analyses suggest that the driver is sexual selection involving sexual conflict, in which escalating male competition benefits females. Male‐only semelparity appears to be consistent with the reproductive effort model, which is based on optimal allocation of reproductive effort and does not require high post‐reproductive mortality risk to favour semelparity.
(4) In animals with male‐only semelparity, we often observe characteristics associated with intense and escalating effort (e.g. mate guarding); traits that trade off survival and future reproduction for current increased paternity.
(5) In spiders, insects, and mammals in which only the males of a species are semelparous, spermatogenic tissue fails permanently at maturity, before the mating period. Males accumulate gametes before sexual maturity, then they release this lifetime store of sperm in a large pulse in their first mating period after maturity. We propose that this convergent phenomenon is an adaptation to sperm competition that maximises the volume of sperm released in a brief mating pulse. This mechanism is analogous to capital breeding in some female animals.

## References

[brv70197-bib-0001] Andrade, M. C. B. (1996). Sexual selection for male sacrifice in the Australian redback spider. Science 271, 70–72.

[brv70197-bib-0002] Andrade, M. C. B. (2003). Risky mate search and male self‐sacrifice in redback spiders. Behavioral Ecology 14, 531–538.

[brv70197-bib-0003] Andrade, M. C. B. & Banta, E. M. (2002). Value of male remating and functional sterility in redback spiders. Animal Behaviour 63, 857–870.

[brv70197-bib-0004] Aronson, R. (1991). Ecology, paleobiology, and evolutionary constraint in the octopus. Bulletin of Marine Science 49, 245–255.

[brv70197-bib-0005] Baer, B. (2005). Sexual selection in *Apis* bees. Apidologie 36, 187–200.

[brv70197-bib-0006] Bell, G. (1980). The costs of reproduction and their consequences. American Naturalist 116, 45–76.

[brv70197-bib-0007] Benton, T. G. & Grant, A. (1999). Elasticity analysis as an important tool in evolutionary and population ecology. Trends in Ecology & Evolution 14, 467–471. 10.1016/s0169-5347(99)01724-3 10542452

[brv70197-bib-0008] Bertolani, R. (2001). Evolution of the reproductive mechanisms in tardigrades‐ a review. Zoologischer Anzeiger 240, 247–252.

[brv70197-bib-0009] Birkhead, T. R. (2000). Promiscuity: An Evolutionary History of Sperm Competition and Sexual Conflict. Faber, London, UK.

[brv70197-bib-0010] Birnie‐Gauvin, K. , Bordeleau, X. , Cooke, S. J. , Davidsen, J. G. , Eldoy, S. H. , Eliason, E. J. , Moore, A. & Aarestrup, K. (2021). Life‐history strategies in salmonids, the role of physiology and its consequences. Biological Reviews 96, 2304–2320. 10.1111/brv.12753 34043292

[brv70197-bib-0011] Bradley, A. J. (1990). Failure of glucocorticoid feedback during breeding in the male red‐tailed phascogale *Phascogale calura* (Marsupialia, Dasyuridae). Journal of Steroid Biochemistry and Molecular Biology 37, 155–163. 10.1016/0960-0760(90)90384-w 2173624

[brv70197-bib-0012] Braithwaite, R. W. & Lee, A. K. (1979). A mammalian example of semelparity. American Naturalist 113, 151–155.

[brv70197-bib-0013] Brown, W. D. & Barry, K. L. (2016). Sexual cannibalism increases male material investment in offspring, quantifying terminal reproductive effort in a praying mantis. Proceedings of the Royal Society B‐Biological Sciences 283, 1–6. 10.1098/rspb.2016.0656 PMC4936037 27358366

[brv70197-bib-0014] Bulmer, M. G. (1985). Selection for iteroparity in a variable environment. American Naturalist 126, 63–71.

[brv70197-bib-0015] Carruth, L. L. , Dores, R. M. , Maldonado, T. A. , Norris, D. O. , Ruth, T. & Jones, R. E. (2000). Elevation of plasma cortisol during the spawning migration of landlocked kokanee salmon (*Oncorhynchus nerka kennerlyi*). Comparative Biochemistry and Physiology. Part C, Pharmacology, Toxicology & Endocrinology 127, 123–131. 10.1016/s0742-8413(00)00140-7 11083023

[brv70197-bib-0016] Casadevall, M. , Sarra‐Alarcon, L. , Delgado, E. & Matallanas, J. (2017). The sexual segregation of the European eel, *Conger conger* (Linnaeus, 1758) (Anguilliformes, Congridae) and female semelparity in the northwest Mediterranean. Journal of Fisheries Research 1, 5–14.

[brv70197-bib-0017] Charlesworth, B. (1994). Evolution in Age‐Structured Populations. Cambridge University Press, Cambridge, UK.

[brv70197-bib-0018] Charnov, E. L. & Schaffer, W. M. (1973). Life history consequences of natural selection‐ Cole's result revisited. American Naturalist 107, 791–793.

[brv70197-bib-0019] Christiansen, J. S. , Praebel, K. , Siikavuopio, S. I. & Carscadden, J. E. (2008). Facultative semelparity in capelin *Mallotus villosus* (Osmeridae) – an experimental test of a life history phenomenon in a sub‐arctic fish. Journal of Experimental Marine Biology and Ecology 360, 47–55.

[brv70197-bib-0020] Clifford, H. (1982). Life cycles of mayflies (Ephemoptera) with special reference to voltinism. Quaestiones Entomologicae 18, 15–90.

[brv70197-bib-0021] Cohn, J. & Christensen, T. (1988). In defense of *Nephila clavipes*, Postmate guarding by the male golden orb weaving spider. Journal of Comparative Psychology 4, 319–325.

[brv70197-bib-0022] Cole, L. C. (1954). The population consequences of life history phenomena. Quarterly Review of Biology 29, 103–137. 10.1086/400074 13177850

[brv70197-bib-0023] Crespi, B. J. & Teo, R. (2002). Comparative phylogenetic analysis of the evolution of semelparity and life history in salmonid fishes. Evolution 56, 1008–1020. 10.1111/j.0014-3820.2002.tb01412.x 12093015

[brv70197-bib-0024] Cushing, J. M. (2020). A bifurcation theorem for Darwinian matrix models and an application to the evolution of reproductive life‐history strategies. Journal of Biological Dynamics 15(sup1), S190–S213. 10.1080/17513758.2020.1858196 33295254

[brv70197-bib-0025] Deshmukh, U. (2015). Suicidal maternal care (matriphagy) in *Stegodyphus sarasinorum* (Arachnida, Eresidae). International Journal of Pharma and Bio Sciences 9, 189–192.

[brv70197-bib-0026] Dickman, C. R. (1994). Evolution of semelparity in male dasyurid marsupials, a critique, and an hypothesis of sperm competition. In Carnivorous Marsupials (ed. J. Carnio ), pp. 19–32. Metro Toronto Zoo, Toronto, Canada.

[brv70197-bib-0027] Dodson, J. J. , Laroche, J. & Lecomte, F. (2009). Contrasting evolutionary pathways of anadromy in euteleostean fishes. American Fisheries Society Symposium 69, 63–77.

[brv70197-bib-0028] Edwards, P. D. , Palme, R. & Boonstra, R. (2016). Seasonal programming, not competition or testosterone, drives stress‐axis changes in a partially‐semelparous mammal. Hormones and Behavior 85, 96–101. 10.1016/j.yhbeh.2016.08.007 27561227

[brv70197-bib-0029] Elgar, M. A. & Schneider, J. M. (2004). Evolutionary significance of sexual cannibalism. Advances in the Study of Behaviour 34, 135–163.

[brv70197-bib-0030] Evans, T. (1998). Offspring recognition by mother crab spiders with extreme maternal care. Proceedings of the Royal Society B‐Biological Sciences 265, 129–134.

[brv70197-bib-0031] Evans, T. G. , Hammill, E. , Kaukinen, K. , Schulze, A. D. , Patterson, D. A. , English, K. K. , Curtis, J. M. R. & Miller, K. M. (2011). Transcriptomics of environmental acclimatization and survival in wild adult Pacific sockeye salmon (*Oncorhynchus nerka*) during spawning migration. Molecular Ecology 20, 4472–4489. 10.1111/j.1365-294X.2011.05276.x 21951593

[brv70197-bib-0032] Finch, C. (1994). Latent capacities for gametogenic cycling in the semelparous invertebrate *Nereis* . Proceedings of the National Academy of Sciences of the United States of America 91, 11769–11770. 10.1073/pnas.91.25.11769 PMC45316 7991533

[brv70197-bib-0033] Finch, C. & Rose, M. (1995). Hormones and the physiological architecture of life history evolution. Quarterly Review of Biology 70, 1–52. 10.1086/418864 7732161

[brv70197-bib-0036] Fisher, D. O. (2005). Population density and presence of the mother are related to natal dispersal decisions in male and female *Antechinus stuartii* . Australian Journal of Zoology 53, 103–110.

[brv70197-bib-0034] Fisher, D. O. & Blomberg, S. P. (2011). Costs of reproduction and terminal investment by females in a semelparous marsupial. PLoS One 6, e15226. 10.1371/journal.pone.0015226 PMC3020937 21249185

[brv70197-bib-0035] Fisher, D. O. , Dickman, C. R. , Jones, M. E. & Blomberg, S. P. (2013). Sperm competition drives the evolution of suicidal reproduction in mammals. Proceedings of the National Academy of Sciences of the United States of America 110, 1710–17914. 10.1073/pnas.1310691110 PMC3816400 24101455

[brv70197-bib-0038] Fisher, D. O. , Double, M. C. & Moore, B. D. (2006 *b*). Number of mates and timing of mating affect offspring growth in the small marsupial *Antechinus agilis* . Animal Behaviour 71, 289–297.

[brv70197-bib-0037] Fisher, D. O. , Double, M. C. , Blomberg, S. P. , Jennions, M. D. & Cockburn, A. (2006 *a*). Post‐mating sexual selection increases lifetime fitness of polyandrous females in the wild. Nature 444, 89–92. 10.1038/nature05206 17080089

[brv70197-bib-0039] Fleming, I. A. & Reynolds, J. D. (2004). Salmonid breeding systems. In Evolution Illuminated, Salmon and their Relatives (eds A. Hendry and S. Stearns ), pp. 264–294. Oxford University Press, Oxford.

[brv70197-bib-0040] Forster, L. M. (1992). The stereotyped behaviour of sexual cannibalism in *Latrodectus hasselti* Thorell (Araneae, Theridiidae) the Australian redback spider. Australian Journal of Zoology 40, 1–11.

[brv70197-bib-0041] Fromhage, L. , Elgar, M. A. & Schneider, J. M. (2005). Faithful without care, the evolution of monogyny. Evolution 59, 1400–1405. 16153026

[brv70197-bib-0042] Futami, K. & Akimoto, S. (2005). Facultative second oviposition as an adaptation to egg loss in a semelparous crab spider. Ethology 111, 1126–1138.

[brv70197-bib-0043] Gadgil, M. & Bossert, W. H. (1970). Life historical consequences of natural selection. American Naturalist 104, 1–24.

[brv70197-bib-0044] Gaschk, J. L. , Del Simone, K. , Wilson, R. S. & Clemente, C. J. (2023). Resting disparity in quoll semelparity, examining the sex‐linked behaviours of wild roaming northern quolls (*Dasyurus hallucatus*) during the breeding season. Royal Society Open Science 10, 221180. 10.1098/rsos.221180 PMC9890097 36756058

[brv70197-bib-0045] Gorokhova, E. & Hansson, S. (2000). Elemental composition of *Mysis mixta* (Crustacea, Mysidacea) and energy costs of reproduction and embryogenesis under laboratory conditions. Journal of Experimental Marine Biology and Ecology 246, 103–123. 10.1016/s0022-0981(99)00173-2 10699221

[brv70197-bib-0046] Grearson, A. , Dugan, A. , Sakmar, T. , Sivitilli, D. , Gire, D. , Caldwell, R. , Niell, C. , Dölen, G. , Wang, Z. & Grasse, B. (2021). The lesser Pacific striped octopus, *Octopus chierchiae*, an emerging laboratory model. Frontiers in Marine Science 8, 753483.

[brv70197-bib-0047] Guillet, S. & Vancassel, M. (2001). Dermapteran life‐history evolution and phylogeny with special reference to the Forficulidae family. Evolutionary Ecology Research 3, 441–447.

[brv70197-bib-0048] Hayes, G. L. T. , Simmons, L. W. , Dugand, R. J. , Mills, H. R. , Roberts, J. D. , Tomkins, J. L. & Fisher, D. O. (2019). Male semelparity and multiple paternity confirmed in an arid‐zone dasyurid. Journal of Zoology 308, 266–273.

[brv70197-bib-0049] Herberstein, M. E. , Gaskett, A. C. , Schneider, J. M. , Vella, N. G. F. & Elgar, M. A. (2005). Limits to male copulation frequency, sexual cannibalism and sterility in St Andrew's cross spiders (Araneae, Araneidae). Ethology 111, 1050–1061.

[brv70197-bib-0050] Hironaka, Y. & Abe, H. (2012). Nesting habits of the Japanese foliage spider, *Cheiracanthium japonicum* (Araneae, Miturgidae), host plant preference based on the physical traits of plant leaves. Journal of Natural History 46, 43–44.

[brv70197-bib-0051] Holldobler, B. (1976). Behavioral ecology of mating in harvester ants (Hymenoptera‐ Formicidae‐ *Pogonomyrex*). Behavioral Ecology and Sociobiology 1, 405–423.

[brv70197-bib-0052] Holleley, C. E. , Dickman, C. R. , Crowther, M. S. & Oldroyd, B. P. (2006). Size breeds success, multiple paternity, multivariate selection and male semelparity in a small marsupial, *Antechinus stuartii* . Molecular Ecology 15, 3439–3448. 10.1111/j.1365-294X.2006.03001.x 16968281

[brv70197-bib-0053] Hruska, K. , Hinch, S. , Healey, M. , Patterson, D. , Larsson, S. & Farrell, A. (2010). Influences of sex and activity level on physiological changes in individual adult sockeye salmon during rapid senescence. Physiological and Biochemical Zoology 83, 663–676. 10.1086/652411 20482369

[brv70197-bib-0054] Hughes, P. W. (2017). Between semelparity and iteroparity, empirical evidence for a continuum of modes of parity. Ecology and Evolution 7, 8232–8261. 10.1002/ece3.3341 PMC5648687 29075446

[brv70197-bib-0055] Hurd, L. (1989). The importance of late season flowers to the fitness of an insect predator, *Tenodera sinensis* Saussure (Orthoptera, Mantidae) in an old field community. Entomologist 108, 223–228.

[brv70197-bib-0056] Ibanez, C. M. , Diaz‐Santana‐Iturrios, M. , Lopez‐Cordova, D. A. , Carrasco, S. A. , Pardo‐Gandarillas, M. C. , Rocha, F. & Vidal, E. A. G. (2021). A phylogenetic approach to understand the evolution of reproduction in coleoid cephalopods. Molecular Phylogenetics and Evolution 155, 106972. 10.1016/j.ympev.2020.106972 33035681

[brv70197-bib-0057] Iguchi, K. & Tsukamoto, Y. (2001). Semelparous or iteroparous, resource allocation tactics in the ayu, an osmeroid fish. Journal of Fish Biology 58, 520–528.

[brv70197-bib-0058] Jellyman, D. J. (2021). An enigma, how can freshwater eels (*Anguilla* spp.) be such a successful genus yet be universally threatened? Reviews in Fish Biology and Fisheries 8, s11160‐02109658.

[brv70197-bib-0059] Jonsson, N. , Jonsson, B. & Hansen, L. P. (1997). Changes in proximate composition and estimates of energetic costs during upstream migration and spawning in Atlantic salmon *Salmo salar* . Journal of Animal Ecology 66, 425–436.

[brv70197-bib-0060] Junghanns, A. , Holm, C. , Schou, M. , Sorensen, A. , Uhl, G. & Bilde, T. (2017). Extreme allomaternal care and unequal task participation by unmated females in a cooperatively breeding spider. Animal Behaviour 132, 101e107.

[brv70197-bib-0061] Kern, C. C. & Gems, D. (2022). Semelparous death as one element of iteroparous aging gone large. Frontiers in Genetics 13, 880343. 10.3389/fgene.2022.880343 PMC9218716 35754809

[brv70197-bib-0062] Kerr, J. B. & Hedger, M. P. (1983). Spontaneous spermatogenic failure in the marsupial mouse *Antechinus stuartii* Macleay (Dasyuridae, Marsupialia). Australian Journal of Zoology 31, 445–466.

[brv70197-bib-0136] Kim, K. W. , Roland, C. & Horel, A. (2000). Functional value of matriphagy in the spider *Amaurobius ferox* . Ethology 106: 729–742.

[brv70197-bib-0063] Kindsvater, H. K. , Braun, D. C. , Otto, S. P. & Reynolds, J. D. (2016). Costs of reproduction can explain the correlated evolution of semelparity and egg size, theory and a test with salmon. Ecology Letters 19, 687–696. 10.1111/ele.12607 27146705

[brv70197-bib-0064] Kirkendall, L. R. & Stenseth, N. C. (1985). On defining breeding once. The American Naturalist 125, 189–204.

[brv70197-bib-0065] Klinkhamer, P. G. L. , Kubo, T. & Iwasa, Y. (2008). Herbivores and the evolution of the semelparous perennial life‐history of plants. Evolutionary Biology 10, 529–550.

[brv70197-bib-0066] Knoflach, B. & Benjamin, S. (2003). Mating without sexual cannibalism in *Tidarren sisyphoides* (Araneae, Theridiidae). Journal of Arachnology 31, 445–448.

[brv70197-bib-0067] Knoflach, B. & Van Harten, A. (2000). Palpal loss, single palp copulation and obligatory mate consumption in *Tidarren cuneolatum* (Tullgren, 1910) (Araneae, Theridiidae). Journal of Natural History 34, 1639–1659.

[brv70197-bib-0068] Knoflach, B. & van Harten, A. (2001). *Tidarren argo* sp nov (Araneae, Theridiidae) and its exceptional copulatory behaviour, emasculation, male palpal organ as a mating plug and sexual cannibalism. Journal of Zoology 254, 449–459.

[brv70197-bib-0069] Kohno, K. (1997). Possible influences of habitat characteristics on the evolution of semelparity and cannibalism in the Hump earwig *Anechura harmandi* . Research in Population Ecology 39, 111–116.

[brv70197-bib-0070] Kuntner, M. , Kralj‐Fiser, S. , Schneider, J. M. & Li, D. (2009). Mate plugging via genital mutilation in nephilid spiders, an evolutionary hypothesis. Journal of Zoology 277, 257–266.

[brv70197-bib-0072] Larsen, L. & Dufour, S. (1993). Growth, reproduction and death in lampreys and eels. In Fish Ecophysiology (Volume 9, eds J. Rankin and F. Jensen ), pp. 72–104. Springer, Dordrecht.

[brv70197-bib-0071] Laumer, C. E. , Fernandez, R. , Lemer, S. , Combosch, D. , Kocot, K. M. , Riesgo, A. , Andrade, S. C. S. , Sterrer, W. , Sorensen, M. V. & Giribet, G. (2019). Revisiting metazoan phylogeny with genomic sampling of all phyla. Proceedings of the Royal Society B‐Biological Sciences 286, 20190831. 10.1098/rspb.2019.0831 PMC6650721 31288696

[brv70197-bib-0073] Lazenby‐Cohen, K. A. & Cockburn, A. (1988). Lek promiscuity in a semelparous mammal, *Antechinus stuartii* (Marsupialia, Dasyuridae)? Behavioral Ecology and Sociobiology 22, 195–202.

[brv70197-bib-0074] Lee, Q. Q. , Oh, J. , Kralj‐Fiser, S. , Kuntner, M. & Li, D. Q. (2012). Emasculation, gloves‐off strategy enhances eunuch spider endurance. Biology Letters 8, 733–735. 10.1098/rsbl.2012.0285 PMC3440970 22696287

[brv70197-bib-0075] LeGrand, R. & Morse, D. (2000). Factors driving extreme sexual size dimorphism of a sit‐and‐wait predator under low density. Biological Journal of the Linnean Society 71, 643–664.

[brv70197-bib-0076] Lennox, R. J. , Nilsen, C. I. , Dahlmo, L. S. , Berhe, S. , Barlaup, B. T. , Normann, E. S. , Landro, Y. , Birnie­Gauvin, K. , Cooke, S. J. & Vollset, K. W. (2024). A comparative study of the alternative life history of iteroparous salmonids. Ecology of Freshwater Fish 33, e12786.

[brv70197-bib-0077] Linde, M. , Donke, E. , Schwartz, S. K. , Hebets, E. A. & Michalik, P. (2026). Extreme sexual size dimorphism correlates with proxies of postcopulatory sexual selection across selected *Dolomedes* fishing spiders. Evolution 80, 1072–1084. 10.1093/evolut/qpag041 41805983

[brv70197-bib-0078] Macqueen, D. J. & Johnston, I. A. (2014). A well‐constrained estimate for the timing of the salmonid whole genome duplication reveals major decoupling from species diversification. Proceedings of the Royal Society B‐Biological Sciences 281, 20132881. 10.1098/rspb.2013.2881 PMC3906940 24452024

[brv70197-bib-0079] McQueen, D. (1978). Field studies of growth, reproduction, and mortality in the burrowing wolf spider *Geolycosa domifex* (Hancock). Canadian Journal of Zoology 56, 2037–2049. 10.1139/z80-150 6775797

[brv70197-bib-0080] Michalik, P. (2009). The male genital system of spiders (Arachnida, Araneae) with notes on the fine structure of seminal secretions. Contributions to Natural History 12, 959–972.

[brv70197-bib-0082] Michalik, P. & Rittschof, C. C. (2011). A comparative analysis of the morphology and evolution of permanent sperm depletion in spiders. PLoS One 6, e16014. 10.1371/journal.pone.0016014 PMC3019211 21264312

[brv70197-bib-0081] Michalik, P. , Knoflach, B. , Thaler, K. & Alberti, G. (2010). Live for the moment – adaptations in the male genital system of a sexually cannibalistic spider (Theridiidae, Araneae). Tissue and Cell 42, 32–36. 10.1016/j.tice.2009.06.004 19643451

[brv70197-bib-0083] Miller, J. A. (2007). Repeated evolution of male sacrifice behavior in spiders correlated with genital mutilation. Evolution 61, 1301–1315. 10.1111/j.1558-5646.2007.00115.x 17542841

[brv70197-bib-0084] Modanu, M. , Michalik, P. & Andrade, M. C. B. (2013). Mating system does not predict permanent sperm depletion in black widow spiders. Evolution and Development 15, 205–212. 10.1111/ede.12034 23607304

[brv70197-bib-0085] Monnin, T. (1998). Monogyny and regulation of worker mating in the queenless ant *Dinoponera quadriceps* . Animal Behaviour 55, 299–306. 10.1006/anbe.1997.0601 9480697

[brv70197-bib-0086] Morita, K. , Tsuboi, J.‐i. , Sahashi, G. , Kikko, T. , Ishizaki, D. , Kishi, D. , Endo, S. & Koseki, Y. (2018). Iteroparity of stream resident masu salmon *Oncorhynchus masou* . Journal of Fish Biology 93, 750–754. 10.1111/jfb.13771 30069884

[brv70197-bib-0087] Morse, D. (1994). Numbers of broods produced by the crab spider *Misumena vatia* (Araneae, Thomisidae). Journal of Arachnology 22, 195–199.

[brv70197-bib-0088] Morse, D. (2012). Reproductive output of a female crab spider, the impacts of mating failure, natural enemies, and resource availability. Entomologia Experimentalis et Applicata 146, 141–148.

[brv70197-bib-0089] Morse, D. & Stephens, E. (1996). The consequences of adult foraging success on the components of lifetime fitness in a semelparous, sit and wait predator. Evolutionary Ecology 10, 361–373.

[brv70197-bib-0090] Murphy, G. I. (1968). Pattern of life history and the environment. American Naturalist 102, 391–403.

[brv70197-bib-0091] Orzack, S. H. & Tuljapurkar, S. (1989). Population dynamics in variable environments. American Naturalist 133, 901–923.

[brv70197-bib-0092] Pandian, T. J. (2018). Reproduction and Development in Echinodermata and Prochordata. CRC Press, Boca Raton, Florida, United States.

[brv70197-bib-0093] Paps, J. , Rossi, M. E. , Bowles, A. M. C. & Álvarez‐Presas, M. (2023). Assembling animals: trees, genomes, cells, and contrast to plants. Frontiers in Ecology and Evolution 11, 1185566.

[brv70197-bib-0094] Persson, L. , Raunsgard, A. , Thorstad, E. B. , Ostborg, G. , Urdal, K. , Saegrov, H. , Ugedal, O. , Hindar, K. , Karlsson, S. , Fiske, P. & Bolstad, G. H. (2023). Iteroparity and its contribution to life‐history variation in Atlantic salmon. Canadian Journal of Fisheries and Aquatic Sciences 80, 577–592.

[brv70197-bib-0095] Pugliese, S. A. (1988). Optimal life history models, effects of nonlinearities in the response of reproductive success to investment. In Biomathematics and Related Computational Problems (ed. L. M. Ricciardi ), pp. 223–235. Springer, Dordrecht.

[brv70197-bib-0096] Quinn, T. P. & Foote, C. J. (1994). The effects of body size and sexual dimorphism on the reproductive behavior of sockeye salmon, *Oncorhynchus nerka* . Animal Behaviour 48, 751–761.

[brv70197-bib-0097] Ramos, M. , Irschick, D. J. & Christenson, T. E. (2004). Overcoming an evolutionary conflict‐ removal of a reproductive organ greatly increases locomotor performance. Proceedings of the National Academy of Sciences of the United States of America 101, 4883–4887. 10.1073/pnas.0400324101 PMC387343 15034176

[brv70197-bib-0098] Rocha, F. , Guerra, A. & Gonzalez, A. (2001). A review of reproductive strategies in cephalopods. Biological Reviews 76, 291–304. 10.1017/s1464793101005681 11569786

[brv70197-bib-0099] Salguero‐Gomez, R. , Jones, O. R. , Jongejans, E. , Blomberg, S. P. , Hodgson, D. J. , Mbeau‐Ache, C. , Zuidema, P. A. , de Kroon, H. & Buckley, Y. M. (2016). Fast‐slow continuum and reproductive strategies structure plant life‐history variation worldwide. Proceedings of the National Academy of Sciences of the United States of America 113, 230–235. 10.1073/pnas.1506215112 PMC4711876 26699477

[brv70197-bib-0100] Salomon, M. , Aflalo, E. , Col, M. & Lubin, Y. (2015). Dramatic histological changes preceding suicidal maternal care in the subsocial spider *Stegodyphus lineatus* (Araneae, Eresidae). Journal of Arachnology 43, 77–85.

[brv70197-bib-0101] Salomon, M. , Schneider, J. & Lubin, Y. (2005). Maternal investment in a spider with suicidal maternal care, *Stegodyphus lineatus* (Araneae, Eresidae). Oikos 109, 614–622.

[brv70197-bib-0102] Sasaki, T. & Iwahashi, O. (1995). Sexual cannibalism in an orb‐weaving spider *Argiope aemula* . Animal Behaviour 49, 1119–1121.

[brv70197-bib-0103] Schaffer, W. & Gadgil, M. (1975). Selection for optimal life histories in plants. In Ecology and Evolution of Communities (eds M. Cody and J. Diamond ), pp. 142–157. Belknap Press, Cambridge, Massachusetts.

[brv70197-bib-0104] Schaffer, W. M. (1974). Optimal reproductive effort in fluctuating environments. American Naturalist 108, 783–790.

[brv70197-bib-0105] Schaffer, W. M. & Elson, P. F. (1975). The adaptive significance of variations in life history among local populations of Atlantic salmon in North America. Ecology 56, 577–590.

[brv70197-bib-0106] Schneider, J. & Fromhage, L. (2010). Monogynous mating strategies in spiders. In Animal Behaviour, Evolution and Mechanisms (ed. P. M. Kappeler ), pp. 441–464. Springer, Berlin.

[brv70197-bib-0107] Schneider, J. M. & Lubin, Y. (1997). Does high adult mortality explain semelparity in the spider *Stegodyphus lineatus* (Eresidae)? Oikos 79, 92–100.

[brv70197-bib-0108] Schneider, J. M. & Michalik, P. (2011). One‐shot genitalia are not an evolutionary dead end‐ regained male polygamy in a sperm limited spider species. BMC Evolutionary Biology 11, 197. 10.1186/1471-2148-11-197 PMC3145602 21740561

[brv70197-bib-0109] Schwartz, S. K. , Wagner, W. E. Jr. & Hebets, E. A. (2013). Spontaneous male death and monogyny in the dark fishing spider. Biology Letters 9, 20130113. 10.1098/rsbl.2013.0113 PMC3730621 23784928

[brv70197-bib-0110] Schwartz, S. K. , Wagner, W. E. Jr. & Hebets, E. A. (2014). Obligate male death and sexual cannibalism in dark fishing spiders. Animal Behaviour 93, 151–156.

[brv70197-bib-0111] Schwartz, S. K. , Wagner, W. E. Jr. & Hebets, E. A. (2016). Males can benefit from sexual cannibalism facilitated by self‐sacrifice. Current Biology 26, 2794–2799. 10.1016/j.cub.2016.08.010 27720621

[brv70197-bib-0112] Shuster, S. (1991). Changes in female anatomy associated with the reproductive moult in *Paracerceis sculpta*, a semelparous isopod crustacean. Journal of Zoology 225, 365–379.

[brv70197-bib-0113] Smith, F. A. & Charnov, E. L. (2001). Fitness trade‐offs select for semelparous reproduction in an extreme environment. Evolutionary Ecology Research 3, 595–602.

[brv70197-bib-0114] Stearns, S. C. (1976). Life history tactics, a review of the ideas. Quarterly Review of Biology 51, 3–47. 10.1086/409052 778893

[brv70197-bib-0115] Stephens, P. A. , Boyd, I. L. , McNamara, J. M. & Houston, A. I. (2009). Capital breeding and income breeding, their meaning, measurement, and worth. Ecology 90, 2057–2067. 10.1890/08-1369.1 19739368

[brv70197-bib-0116] Stockley, P. , Franco, C. , Claydon, A. J. , Davidson, A. , Hammond, D. E. , Brownridge, P. J. , Hurst, J. L. & Baynon, R. J. (2020). Revealing mechanisms of mating plug function under sexual selection. Proceedings of the National Academy of Sciences of the United States of America 2020(117), 27465–27473. 10.1073/pnas.1920526117 PMC7959524 33077605

[brv70197-bib-0117] Suzuki, S. (2022). Brood size manipulation reveals cost on the size of the second clutch in the earwig *Anisolabis maritime* . Behaviour 159, 259–269.

[brv70197-bib-0118] Suzuki, S. , Kitamura, M. & Matsubayashi, K. (2005). Matriphagy in the hump earwig, *Anechura harmandi* (Dermaptera, Forficulidae), increases the survival rates of the offspring. Journal of Ethology 23, 211–213.

[brv70197-bib-0119] Taggart, D. A. & Templesmith, P. D. (1991). Transport and storage of spermatozoa in the female reproductive tract of the brown marsupial mouse, *Antechinus stuartii* (Dasyuridae). Journal of Reproduction and Fertility 93, 97–110. 10.1530/jrf.0.0930097 1920303

[brv70197-bib-0120] Tesar, D. , Kaitala, V. & Ranta, E. (2001). Stochasticity and spatial coexistence of semelparity and iteroparity as life histories. Evolutionary Ecology 15, 193–204.

[brv70197-bib-0121] Tizo‐Pedroso, E. & Del‐Claro, K. (2005). Matriphagy in the neotropical pseudoscorpion *Paratemnoides nidificator* (Balzan 1888) (Atemnidae). Journal of Arachnology 33, 873–877.

[brv70197-bib-0122] Todd, C. , Hadfield, M. & Snedden, W. (1997). Juvenile mating and sperm storage in the tropical corallivorous nudibranch *Phestilla sibogae* . Invertebrate Biology 116, 322–330.

[brv70197-bib-0123] Toyama, M. (1999). Adaptive advantages of maternal care and matriphagy in a foliage spider, *Chiracanthium japonicum* (Araneae, Clubionidae). Journal of Ethology 17, 33–39.

[brv70197-bib-0124] Tripathi, R. , Jangid, A. , Siliwal, M. & Dutta, S. (2020). The first report of matriphagy in *Stegodyphus pacificus* Pocock 1900. Acta Arachnologica 69, 17–21.

[brv70197-bib-0125] Trumbo, S. T. (2013). Maternal care, iteroparity and the evolution of social behavior, a critique of the semelparity hypothesis. Evolutionary Biology 40, 613–626.

[brv70197-bib-0126] Tsukamoto, K. , Chow, S. , Otake, T. , Kurogi, H. , Mochioka, N. , Miller, M. , Aoyama, J. , Kimura, S. , Watanabe, S. , Yoshinaga, T. , Shinoda, A. , Kuroki, M. , Oya, M. , Watanabe, T. , Hata, K. , Ijiri, S. , Kazeto, Y. , Nomura, K. & Tanaka, H. (2011). Oceanic spawning ecology of freshwater eels in the western north Pacific. Nature Communications 2, 179. 10.1038/ncomms1174 PMC3105336 21285957

[brv70197-bib-0127] Unwin, M. , Kinnison, M. & Quinn, T. (1999). Exceptions to semelparity, postmaturation survival, morphology, and energetics of male chinook salmon (*Oncorhynchus tshawytscha*). Canadian Journal of Fisheries and Aquatic Sciences 56, 1172–1181.

[brv70197-bib-0128] Varpe, O. & Ejsmond, M. (2018). Semelparity and iteroparity. In The Natural History of the Crustacea‐ Life Histories (eds M. Thiel and G. Wellborn ), pp. 97–124. Oxford University Press, Oxford, UK.

[brv70197-bib-0129] Wang, Z. & Ragsdale, C. (2018). Multiple optic gland signaling pathways implicated in octopus maternal behaviors and death. Journal of Experimental Biology 221, jeb185751. 10.1242/jeb.185751 PMC6198452 30104305

[brv70197-bib-0130] Wodinsky, J. (1977). Hormonal inhibition of feeding and death in octopus, control by optic gland secretion. Science 198, 948–951. 10.1126/science.198.4320.948 17787564

[brv70197-bib-0131] Woollard, P. (1971). Differential mortality of *Antechinus stuartii* (Macleay), nitrogen balance and somatic changes. Australian Journal of Zoology 19, 347–353.

[brv70197-bib-0133] Young, T. P. (1981). A general model of comparative fecundity for semelparous and iteroparous life histories. American Naturalist 118, 27–36.

[brv70197-bib-0134] Young, T. P. & Augspurger, C. K. (1991). Ecology and evolution of long‐lived and semelparous plants. Trends in Ecology & Evolution 6, 285–289. 10.1016/0169-5347(91)90006-J 21232483

[brv70197-bib-0135] Zeineddine, M. & Jansen, V. A. A. (2009). To age, to die, parity, evolutionary tracking and Cole's paradox. Evolution 63, 1498–1507. 10.1111/j.1558-5646.2009.00630.x 19492994

